# eDNA metabarcoding as a new surveillance approach for coastal Arctic biodiversity

**DOI:** 10.1002/ece3.4213

**Published:** 2018-07-13

**Authors:** Anaïs Lacoursière‐Roussel, Kimberly Howland, Eric Normandeau, Erin K. Grey, Philippe Archambault, Kristy Deiner, David M. Lodge, Cecilia Hernandez, Noémie Leduc, Louis Bernatchez

**Affiliations:** ^1^ St. Andrews Biological Station (SABS) Fisheries and Oceans Canada St. Andrews NB Canada; ^2^ Central and Arctic Region Fisheries and Oceans Canada Freshwater Institute Winnipeg MB Canada; ^3^ Department of Biology Institut de Biologie Intégrative et des Systèmes (IBIS) Université Laval Québec QC Canada; ^4^ Division of Science, Mathematics and Technology Governors State University University Park IL USA; ^5^ Department of Biology Université Laval Québec QC Canada; ^6^ Department of Evolutionary Biology and Environmental Studies University of Zurich Zürich Switzerland; ^7^ Department of Ecology and Evolutionary Biology Cornell University Ithaca NY USA

**Keywords:** Arctic, coastal biodiversity, eDNA metabarcoding, global changes, invasion, spatio‐temporal distribution

## Abstract

Because significant global changes are currently underway in the Arctic, creating a large‐scale standardized database for Arctic marine biodiversity is particularly pressing. This study evaluates the potential of aquatic environmental DNA (eDNA) metabarcoding to detect Arctic coastal biodiversity changes and characterizes the local spatio‐temporal distribution of eDNA in two locations. We extracted and amplified eDNA using two COI primer pairs from ~80 water samples that were collected across two Canadian Arctic ports, Churchill and Iqaluit, based on optimized sampling and preservation methods for remote regions surveys. Results demonstrate that aquatic eDNA surveys have the potential to document large‐scale Arctic biodiversity change by providing a rapid overview of coastal metazoan biodiversity, detecting nonindigenous species, and allowing sampling in both open water and under the ice cover by local northern‐based communities. We show that DNA sequences of ~50% of known Canadian Arctic species and potential invaders are currently present in public databases. A similar proportion of operational taxonomic units was identified at the species level with eDNA metabarcoding, for a total of 181 species identified at both sites. Despite the cold and well‐mixed coastal environment, species composition was vertically heterogeneous, in part due to river inflow in the estuarine ecosystem, and differed between the water column and tide pools. Thus, COI‐based eDNA metabarcoding may quickly improve large‐scale Arctic biomonitoring using eDNA, but we caution that aquatic eDNA sampling needs to be standardized over space and time to accurately evaluate community structure changes.

## 1 INTRODUCTION

In the Arctic, climate change and marine invasions are expected to result in over 60% species turnover from present biodiversity with substantial impacts on marine ecosystems (Cheung et al., 2009). Climate change is opening new waterways in the Arctic Ocean, resulting in greater shipping traffic (ACIA 2004; Arctic Council 2009; Guy & Lasserre, 2016). Predicted increases in shipping frequency and routes (Eguíluz, Fernández‐Gracia, Irigoien, & Duarte, 2016; Miller & Ruiz, 2014; Smith & Stephenson, 2013), increased infrastructure development in ports (Gavrilchuk & Lesage, 2014), and associated chemical/biological pollution will place other ecosystem services at risk. Furthermore, the introduction of nonindigenous species (NIS) may displace native species, alter habitat and community structure and increase aquaculture and fishing gear fouling in estuaries and coastal zones (Goldsmit et al., 2018; Grosholz, 2002; Parker et al., 1999). Currently, the continuous monitoring needed to evaluate large‐scale changes in coastal biodiversity and faunal assemblages in the Canadian Arctic is limited (Archambault et al., 2010), hindering risk management and ecosystem sustainability planning (Larigauderie et al., 2012).

Recent advances in the collection and analysis of environmental DNA (eDNA) provide a new complementary approach that can help to fill gaps in regional species distribution data left by logistically difficult traditional methods (e.g., bottom trawl, SCUBA diving) (Deiner et al., 2017), particularly in remote and otherwise challenging locations. eDNA allows for the detection of traces of DNA in water from macro‐organisms (Thomsen, Kielgast, Iversen, Wiuf, et al., 2012). Collecting water samples for eDNA surveys could allow rapid sample collection, reduce the cost associated with data collection/shipping, and is less destructive because it does not require the manipulation of organisms (Lodge et al., 2012; Taberlet, Coissac, Hajibabaei, & Rieseberg, 2012). eDNA metabarcoding (i.e., high‐throughput eDNA sequencing) can enable the identification of millions of DNA fragments/sample, providing a powerful approach to survey aquatic biodiversity. Repeated eDNA surveys could potentially be used to evaluate long‐term biodiversity changes such as detecting native species loss and declines, NIS introductions and range expansions, and community structure changes. However, the detection of species using eDNA varies as a function of the population densities (Lacoursière‐Roussel, Côté, Leclerc, & Bernatchez, 2016; Lacoursière‐Roussel, Dubois, & Bernatchez, 2016; Mahon et al., 2013), life history traits, shedding rates (Lacoursière‐Roussel, Rosabal, & Bernatchez, 2016; Sassoubre, Yamahara, Gardner, Block, & Boehm, 2016) local environmental conditions and technical approaches such as sequencing efforts and primer biases (Freeland, 2017; Pawluczyk et al., 2015). Moreover, major concerns with eDNA metabarcoding, including its ability to accurately identify sequences to species (Chain, Brown, MacIsaac, & Cristescu, 2016) and the unknown ecological dynamics of eDNA in coastal ecosystems, need to be studied before marine biodiversity can be compared across spatial and temporal scales using this method.

Little is currently known about the efficacy of eDNA metabarcoding in surveying long‐term variation in marine coastal biodiversity (Lim et al., 2016; Port et al., 2016; Thomsen & Willerslev, 2015). Relative to freshwater ecosystems where more studies have been conducted, eDNA in coastal marine ecosystems is diluted into a much larger volume of water and exposed to pronounced hydrodynamics (e.g., tides, currents) and variation in abiotic conditions (e.g., salinity, temperature), which is likely to affect eDNA transport and degradation (Foote et al., 2012; Thomsen, Kielgast, Iversen, Møller, et al., 2012). In spite of these challenges, a recent study of horizontal spatial eDNA distribution in the Puget Sound (Washington, USA; O’Donnell et al., 2017) was successful in revealing fine scale distribution of species in these communities. In Arctic ecosystems, higher eDNA transport and diffusion is expected due to slower DNA degradation in cold‐water temperatures, but no study has yet characterized aquatic eDNA distribution in this environment. Improving our understanding of the ecology of eDNA—the myriad of interactions between extraorganismal genetic material and its environment (Barnes & Turner, 2016)—in various ecosystems is fundamental to determining how eDNA can and cannot improve biodiversity research.

Our objective is to explore the potential of eDNA as a biodiversity monitoring approach to assist in rapid detection of coastal biodiversity shifts on large spatial scale in two Arctic coastal areas: Churchill and Iqaluit. These two Arctic commercial ports are expected to be particularly prone to biodiversity changes because they are among the top three ports in the Canadian Arctic with respect to vessel arrivals and associated ballast and/or hull fouling invasions risk (Chan, Bailey, Wiley, & MacIsaac, 2013). More specifically, we estimate the proportion of the Arctic biodiversity that can be identified at the species level with eDNA, and we then characterize the spatio‐temporal distribution of eDNA with respect to water column depths, tide pools, and seasons.

## 2 MATERIALS AND METHODS

**Figure 1:**
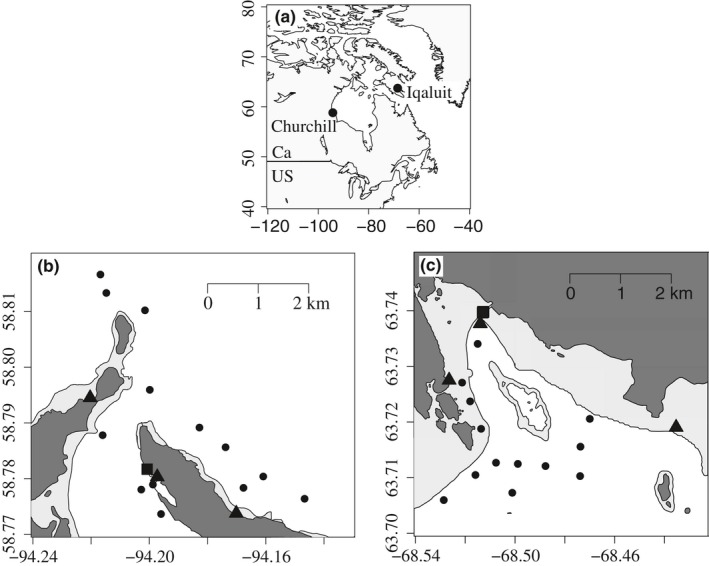
Geographical locations of the sampling port in the Canadian Arctic (map a) and the site distribution within Churchill (map b) and Iqaluit (map c). Subtidal areas are shown in white and the intertidal areas in light gray. Circles depict the water column sites, triangles are the tide pools sites and the squares are the S20 and F20 shore sampling sites

The spatio‐temporal eDNA distribution was characterized at three different depths in the water column, in tide pools, and between summer and fall seasons. Specifically, water samples were collected in 13 subtidal sites at three different depths (surface, mid‐depth and deep water (i.e., 50 cm from the bottom), 12 tide pool sites within three intertidal areas (*N* = 4 sites/area) and 20 samples were collected at a single site from the shore approximately 2 m spaced along a transect (Figure 1). For the summer period (without ice cover), Churchill and Iqaluit were surveyed in 2015 between August 11–14 and August 17–22, respectively (hereafter called S20). To evaluate seasonal effects (Iqaluit only), the 20 samples at a single site were collected during fall (November 18, 2015) near shore from water that rose between ice pans at high tide (hereafter called F20).

Each sample (250 ml water) was collected using a Niskin bottle and then rapidly filtered in the field through a 0.7 μm glass microfiber filter (Whatman GF/F, 25 mm) using syringes (BD 60 ml; Kranklin Lakes, NJ, USA). Field negative controls (i.e., 250 ml distilled water) were filtered for every 10 samples. Filters were preserved at 4°C in 700 μl of Longmire’s lysis/preservation buffer within a 2 ml tube for up to 3 weeks (Wegleitner, Jerde, Tucker, Chadderton, & Mahon, 2015) and then frozen at −20°C until DNA extraction. To reduce risk of cross‐contamination during sampling and the filtration process, individual sampling kits were used for each sample (bottles and filter housing sterilized with a 10% bleach solution and new sterilized gloves, syringes, and tweezers). Each sampling kit was exposed to UV for 30 min. To reduce the risk of laboratory cross‐contamination, procedures for eDNA extraction, PCR preparation, and post‐PCR steps were all performed in different rooms and PCR manipulations were performed in a decontaminated UV hood. Samples from a specific port were all treated together, and the bench space and laboratory tools were bleached and exposed to UV for 30 min prior to processing the next port. Sites within a port were processed in a randomized order.

### 2.1 eDNA extraction, amplification and sequencing

DNA was extracted using a QIAshredder and phenol/chloroform protocol (see Supporting Information Appendix S1). Negative control extractions (950 μl distilled water) were performed for each sample batch (i.e., one for each 23 samples). Two pairs of universal metazoan mitochondrial cytochrome c oxidase subunit I (COI) primers that have been developed and tested on a broad array of marine species were used to amplify eDNA from as many metazoan taxa as possible: the forward mlCOIintF (Leray et al., 2013) and reverse jgHCO2198 (Geller, Meyer, Parker, & Hawk, 2013) amplifying 313 bp (hereafter called COI1) and the forward LCO1490 (Folmer, Black, Hoeh, Lutz, & Vrijenhoek, 1994) and reverse ill_C_R (Shokralla et al., 2015) amplifying 325 bp (COI2).

The performance of the two selected primer pairs used in this study was previously tested on 104 zooplankton species and was validated on mock metazoan communities collected in Canadian ports by Zhang (2017). Based on a total of 13 COI primer pairs selected from the literature and tested, Zhang (2017) showed the efficiency of using multiple COI primer pairs in a single Illumina run to recover species by metabarcoding and detected 32% of species using COI1 and 49% of species using COI2. Here, the DNA amplification protocols for both primer pairs were optimized in vitro using 12 Arctic specimens and 12 potential invaders (i.e., annealing temperature gradient using DNA extracted from tissue samples; Supporting Information Table S1). The primer sequences and sequence databases were also evaluated in silico for their ability to detect native and potential nonindigenous Arctic metazoans. A list of recorded coastal Arctic metazoans was obtained by pooling all Arctic species databases that we had access to (*N* total = 897 metazoan identified at the species level; Fisheries and Oceans Canada Arctic Marine Invertebrate Database (Supporting Information Appendix S2), Archambault unpublished data, Cusson, Archambault, and Aitken (2007), Goldsmit, 2016; Goldsmit, Howland, & Archambault, 2014; K. Howland, P. Archambault, N. Simard and R Young, unpublished data, Piepenburg et al., 2011; Link, Piepenburg, & Archambault, 2013; López, Olivier, Grant, & Archambault, 2016; Olivier, San Martín, & Archambault, 2013; Roy, Iken, & Archambault, 2015; Young, Abbott, Therriault, & Adamowicz, 2016). Potential NIS invaders (*N* = 130 species) were targeted based on (1) screening level risk assessments and predictive species distribution models indicating they were high risk (Goldsmit et al., 2018), (2) their presence in ports connected to the Canadian Arctic, and/or (3) their presence in ballast waters and hulls of ships based on monitoring at Canadian Arctic ports (Chan, MacIsaac, & Bailey, 2015; Chan et al., 2012). Historical data include many Arctic regions, surveyed mainly during the open water period, with focal taxa varying among surveys. Comprehensive port surveys in Churchill and Iqaluit were only conducted once every few years (Churchill 2007, 2011 and 2015; Iqaluit 2012 and 2015–2016). A script was used to determine whether the primer sequences were present for the targeted species (species previously recorded from the Artic and potential NIS) available in the NCBI and BOLD databases (September 2016; http://www.barcodinglife.org). Searches for Arctic species in the sequence databases were performed with Python and Bash programs (developed by Jérôme Laroche at the Institut de Biologie Intégrative et des Systèmes (IBIS), Université Laval) and analyses are freely available on Bitbucket (https://bitbucket.org/jerlar73/env-dna).

Three PCR replicates were performed for each eDNA sample and each primer set. DNA amplifications were performed in a one‐step dual‐indexed PCR approach designed for Illumina instruments at IBIS. The final reaction volume for each PCR replicate was 24 μl; including 12.5. μl Qiagen Multiplex Mastermix, 6.5 μl diH_2_0, 1 μl of each primer (10 μM), and 3.0 μl of DNA. For all samples, the PCR mixture was denatured at 95°C for 15 min, followed by 35 cycles (94°C for 30 s, 54°C for 90 s (except for the COI2 primers, which were at 52°C for 90 s and 72°C for 60 s) and a final elongation at 72°C for 10 min. Products of the three aliquots were pooled for each sample. A negative PCR control was performed for each sample and primer set. All amplifications were visualized on a 1.5% agarose gel electrophoresis. No positive amplification of the PCR negative control was observed. Field and extraction negative controls were treated exactly the same as regular samples and were also sequenced. Pooled products were purified using Axygen PCR clean up kit following the manufacturer’s recommended protocol. Libraries were quantified by AccuClear Ultra High Sensitivity dsDNA Quantification Kit using the TECAN Spark 10 M Reader for each sample and were pooled in equal molar concentrations to maximize equal sequence depth per sample location (150 and 37 ng per sample for COI1 and COI2 primer sets, respectively, in Churchill and 200 and 300 ng per sample for COI1 and COI2 primer sets, respectively, in Iqaluit). When Quant‐iT PicoGreen (Life Technologies) did not detect any DNA, 22.0 μl PCR mixtures were mixed nonetheless (see Supporting Information Table S2 for the concentration and volume for each sample separately).

Sequencing was carried out using an Illumina MiSeq (Illumina, San Diego, CA, USA) using a paired‐end MiSeq Reagent Kit V3 (Illumina) and following the manufacturer’s instructions (Supporting Information Appendix S1). Each port was analyzed on a separate run to ensure independency, but the samples within a port were pooled within a single Illumina MiSeq run to ensure the equality of sequencing depth among samples. Raw sequences reads were deposited in NCBI’s Sequence Read Archive (SRA, http://www.ncbi.nlm.nih.gov/sra) under Bioproject PRJNA388333.

### 2.2 Taxonomic identification

Forward and reverse sequences for each sample were trimmed using Trimmomatic 0.30 (Bolger, Lohse, & Usadel, 2014). FastQC version v0.11.3 was used to confirm the quality of the trimmed reads (Andrews, 2010). The Fastq quality scores were all well above 20 for the trimmed reads. Reads were then merged using FLASH v1.2.11 with a minimum overlap of 30 bp (Magoč & Salzberg, 2011). “Orphan” reads with <30 bp of overlap between forward and reverse reads were discarded and only merged reads were used in the analyses. COI1 and COI2 amplicons were split using a Python script which searches for degenerate primers at the beginning and end of each sequence and only keeps sequences where there is positive identification for both primers ≥270 bp. These sequences were compared for identity with the metazoan sequences present in the Barcode of Life Database (BOLD) (Ratnasingham & Hebert, 2007; available on the BOLDSYSTEM S3 website, http://www.boldsystems.org, on the 22nd August 2016). Terrestrial species (insects, human, birds, and mammals) and sequences that did not have a taxonomic name assigned at the species level were removed from the reference database.

To examine biodiversity at the species level, direct taxonomic assignment of each merged read with ≥97% identity was performed using the *Barque* pipeline version 0.9 (see Supporting Information Appendix S3), an open source and freely available metabarcoding analysis pipeline (http://www.github.com/enormandeau/barque). Reads matching with equal quality scores to more than one species due to low interspecific divergence were found using usearch. Only 156 reads (i.e., 0.02% reads, 17 cases) in total were found with such multiple hits. For each case, the list of species was scrutinized and species that were clearly not expected in the Arctic based on Ocean Biogeographic Information system (OBIS), The World Porifera Database, the World Register of Marine Species (WoRMS) database, invasion risk assessments (see references above and Supporting Information Appendix S2), and expert knowledge were removed from the sequence reference database mentioned above (see Supporting Information Table S3 for details about the multiple hits and actions made for each species). The pipeline was run again to find the top hits only. The proportion of missing species assignments due to BOLD incompleteness was further explored for each metazoan phyla using Operational Taxonomic Units (OTU) clustering according to 97% similarity with swarm 2.2.0 (Mahé, Rognes, Quince, De Vargas, & Dunthorn, 2015; see bioinformatic details Supporting Information Appendix S3). OTUs represented by a single read (singletons) were excluded and the identity between the representative sequences and the BOLD database was performed using vsearch (Rognes, Flouri, Nichols, Quince, & Mahé, 2016). For each phylum, proportion of the biodiversity assigned to the species level was obtained from the number of OTUs between 97–100% (similar to threshold used to assign species for sequences in the BOLD database) relative to those between 80–97% (i.e., below species level).

### 2.3 Statistical analyses

Sampling effort is an important factor to consider in both traditional and eDNA biodiversity surveys. Two levels of port‐specific sampling effort were explored: number of unique species per read (a measure of sequencing effort) and the number of unique species per sample (a measure of eDNA collection effort). For water column (surface, mid‐depth and deep), tide pool and shore (S20 and F20) sampling locations, we plotted both read and sample rarefied accumulation curves to visualize whether or when a plateau was reached (which would indicate adequate sequencing and sampling effort to characterize all species). We also inspected the relative position of the read curve compared to the sample curve, as read curves lying above sample curves typically indicate spatial aggregation of species (Gotelli & Colwell, 2010), or in this case eDNA sequences. These sampling effort analyses were performed in R 3.4.1 using the *specaccum* function in the *vegan* package.

All further statistical analyses were performed using R 3.0.3. The spatial distribution of eDNA and the seasonal variability in the community composition was represented using Principal component analysis (PCoA) and tested using PERMANOVA (Anderson, 2001) after Hellinger transformation. Hellinger transformation was appropriate to deal with the large proportion of zeros and reduces the importance of large abundances (Legendre & Legendre, 1998) that could be due to the eDNA origin (e.g., capture of cell or mitochondria vs. extracellular DNA) or the amplification process. Species that mostly contributed to the dissimilarity/similarity between the treatments (depths and tide pools vs. water column) were identified using SIMPER analysis using the *simper()* function of the *vegan* package. Shannon diversity indices were calculated with the R package vegan. Analyses of variance (ANOVAs) were used to test whether species diversity, richness and log_10_(reads abundance) varied as a function of sampling location (i.e., water column and tide pools; sites included as a random variable) and water depths for each port separately using the *lme()* function of the *NLME* package (Pinheiro, Bates, DebRoy, & Sarkar, 2017) with sites included as a random variable (interactions between sites and depths could not be tested due to unique values per depth). The seasonal effect on read abundance (i.e., metazoan reads, see section *taxonomic identification*), Shannon diversity and species richness was evaluated using a Student’s *t* test comparing the S20 and F20 samples in Iqaluit. Sørensen and Jaccard nonparametric estimates were calculated for location pairs using the *SimilarityPair* function of the *SpadeR* package in R (Chao, Ma, Hsieh, & Chiu, 2016) to test for the level of similarity in species composition between sampling locations and seasons.

## 3 RESULTS

After bioinformatics filtering (Supporting Information Table S2), we obtained 712,494 aquatic eukaryotic reads in Churchill (200,732 reads for COI1 and 511,762 reads for COI2) and 178,728 reads in Iqaluit (100,139 reads for COI1 and 78,589 reads for COI2). No amplification was visualized on the gel electrophoresis for the negative PCR controls and no significant eDNA reads were sequenced in any of the negative extractions controls (Churchill: 1–12 reads, average of 0.05% of the eDNA sample reads; Iqaluit: 1–8 reads, average of 0.17% of the eDNA samples reads) or the negative field controls (Churchill: 2–73 reads, 0.30% in average of the eDNA sample reads; Iqaluit: 0–54 reads, 0.75% in average of the eDNA sample reads).

**Figure 2:**
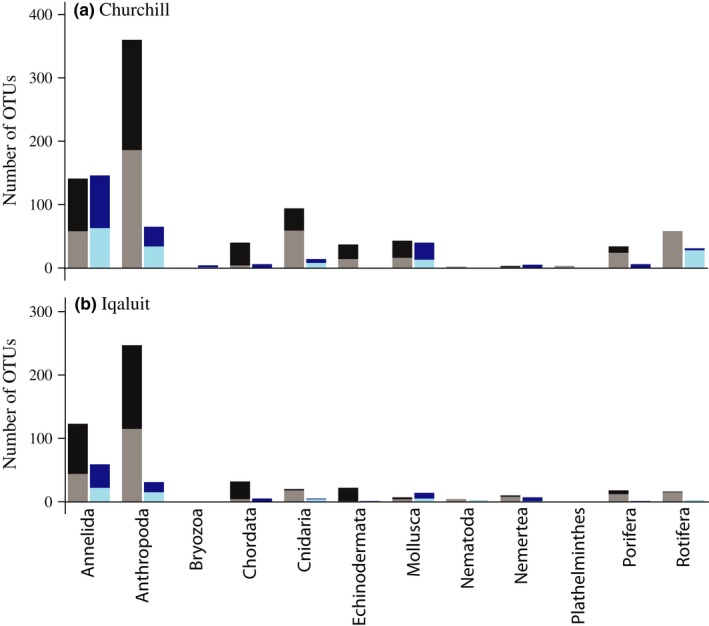
The number of Operational taxonomic units (OTU) identified at the species level (dark: ≥97% identity) relative to those identified below the species level (lighten: ≥85% and <97% identity) for each phylum and from the COI1 (mlCOIintF‐jgHCO2198: black and gray) and COI2 (LCO1490‐ill_C_R: blue) primer sets separately for both Arctic sampling ports (Churchill and Iqaluit)

Cytochrome c oxidase subunit I sequences of 46% and 44% of the known Canadian Arctic native taxa and 63% and 53% of potential invaders are currently in GenBank or BOLD database, respectively. In parallel, the proportion of OTUs matched to a species in the eDNA survey was 53% in Churchill and 50% in Iqaluit (see the proportion by phylum in Figure 2). For both ports, the sampling effort could have been increased to reveal additional species as the sample and read accumulation curves did not plateau (Supporting Information Figure S1). However, there was little evidence for spatial eDNA aggregation within a location as sample‐based curves fell only slightly below read curves, and within 95% confidence intervals, at all locations.

### 3.1 Taxonomic composition in Arctic coastal ports

A total of 181 species were detected in the eDNA survey; 140 species in Churchill and 87 species in Iqaluit (see Supporting Information Figure S2 for the species list for each primer set and their status according to previous Canadian Arctic reports). Forty‐eight species were amplified with both COI primer sets, 116 species recorded by the COI1 primer set only and 17 species by the COI2 primer set. At the species level, the primer sets detected a total of ten phyla; including nine phyla for the COI1 primer set (44 Annelida species, 31 Arthropoda, 35 Chordata, 17 Cnidaria, 17 Echinodermata, eight Mollusca, three Nemertea, five Porifera and four Rotifera) and 10 for the COI2 primer set (27 Annelida species, ten Arthropoda, two Bryozoa, five Chordata, six Cnidaria, one Echinodermata, eight Mollusca, two Nemertea, three Porifera and one Rotifera). In contrast to mock metazoan communities (see method section), a larger number of species was identified using COI1 primers than COI2 primers, but the latter detected proportionately more Annelida and Porifera.

For both ports, 74.0% of the species detected have been previously reported from the Arctic (Churchill: 70.0% and Iqaluit: 87.4%; COI1: 78.6% and COI2: 61.5%). The number of species detected using eDNA in Churchill and Iqaluit represents 10.9% and 8.5% metazoan species recorded within the overall Arctic species databases. Forty‐seven species not previously reported were detected, including 15 Annelida, five Arthropoda, two Bryozoa, four Chordata, eight Cnidaria, two Echinodermata, four Mollusca, three Nemertea and four Rotifera species. The only potential invaders detected, the Arthropoda *Acartia tonsa*, was found with the COI1 primers in Churchill (64 reads averaging 99.4% identity with the sequence references). This species was previously recorded in ballast water in ports connected to Churchill and is considered a potential invader (Chan et al., 2012). However, COI sequences in BOLD assigned to *A. tonsa* are not monophyletic and several are indistinguishable from sequences assigned to the native *A. hudsonica*, suggesting misidentification of some *Acartia* specimens in BOLD.

### 3.2 Spatial eDNA distribution

**Figure 3:**
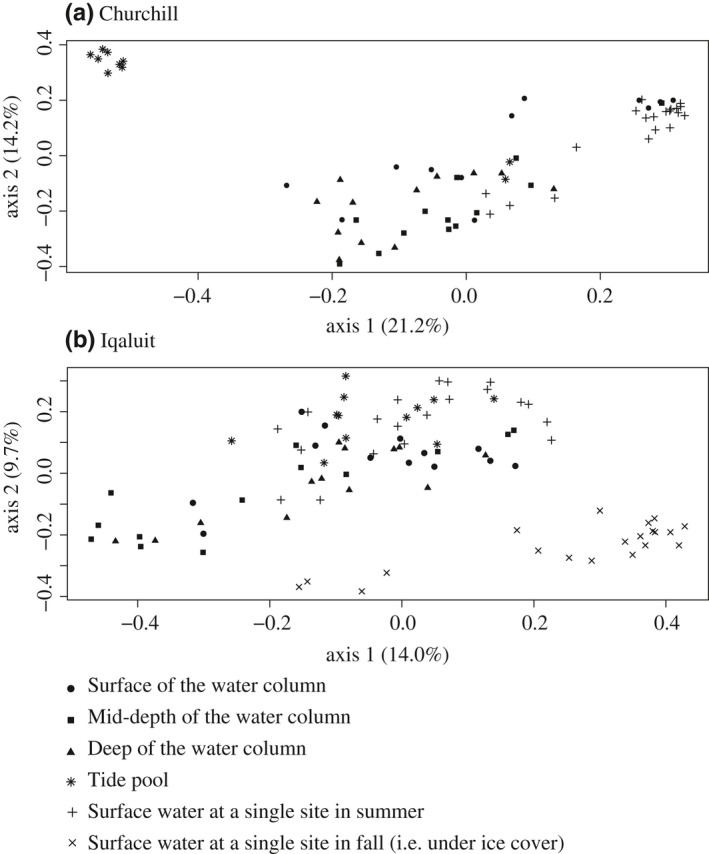
Principal component analysis depicting the community structure at the species level among sampling locations: water column (surface, mid‐depth and deep water), tide pools (i.e., intertidal zone) and surface water collected in a single site in summer (i.e., S20) and in fall (F20) for both Arctic sampling ports (Churchill and Iqaluit). Ports were analyzed separately because each port was treated on a separate sequencing run

**Figure 4:**
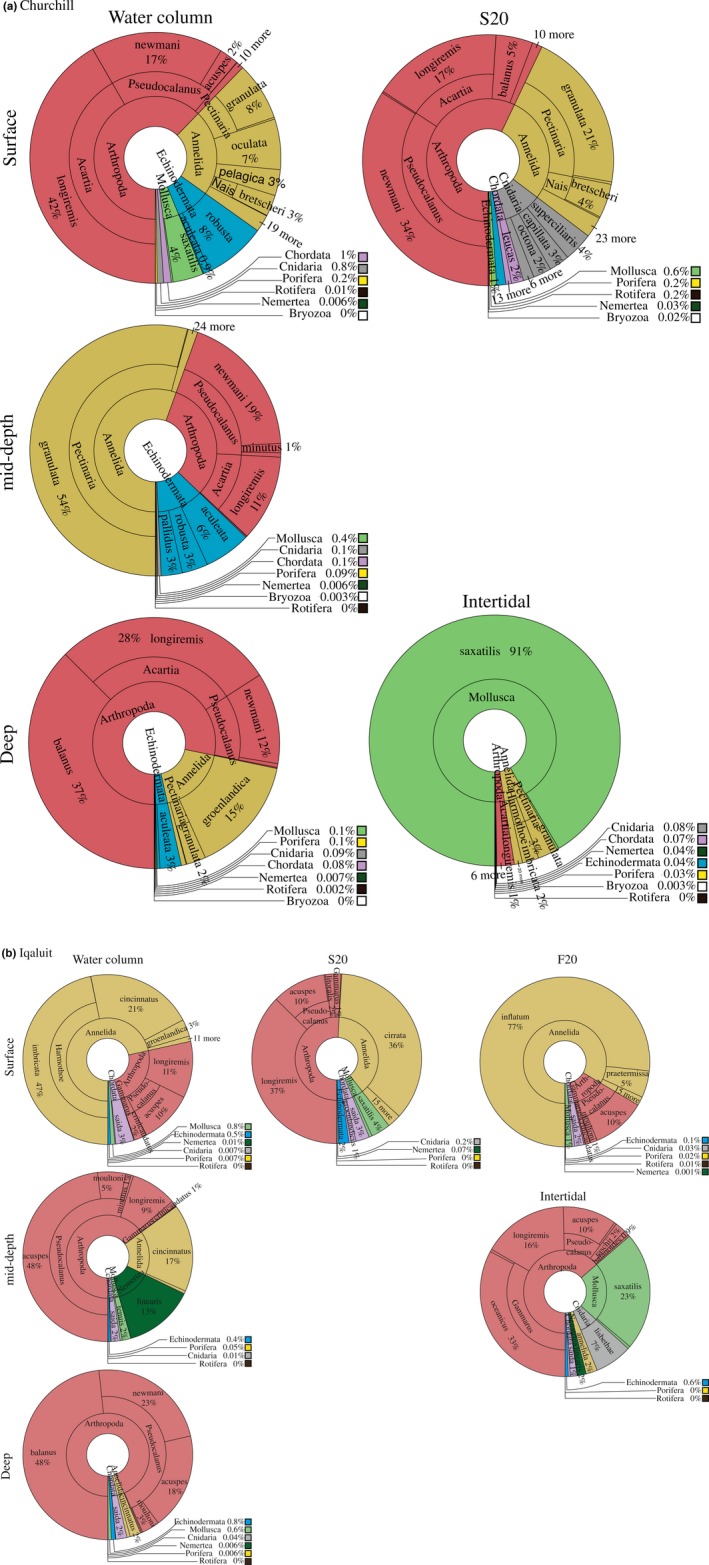
eDNA community differences between sampling locations (i.e., water column (surface, mid‐depth and deep), tide pools) and seasons (summer S20 and Fall F20). The different layers represent phyla (central), genus and species (peripheral)

For both ports, the community structure differed significantly between the water column and the tide pools, but the proportion of explained variance was greater for Churchill than Iqaluit (Figure 3, PERMANOVA; Churchill: *R* ^2^ = 0.21, *p *<* *0.001; Iqaluit: *R* ^2^ = 0.12, *p *<* *0.001; seasonality did not impact analysis of spatial variability when analyzed separately). For both ports, the water column was dominated by Arthropoda (Churchill: 91,219 reads for COI1 and 164,080 reads for COI2; Iqaluit: 30,550 reads for COI1 and 16,971 reads for COI2), followed by Annelida (Churchill: 28,607 reads for COI1 and 110,643 reads for COI2; Iqaluit: 11,518 reads for COI1 and 2,621 reads for COI2) (Figure 4). Mollusca species were mainly detected in tide pools at both ports (91% and 23%, respectively, for Churchill and Iqaluit; Figure 4), and were by far the dominant taxa in Churchill with the majority being *Littorina saxatilis* for COI1 and COI2 (95.8% (i.e., 14,219 reads) and 100% (i.e., 198,684 reads) of Mollusca reads; cumulative contributions for Churchill = 62.4% and Iqaluit = 52.0%); tide pools were dominated by Arthropoda species in Iqaluit (Figure 4).

**Figure 5:**
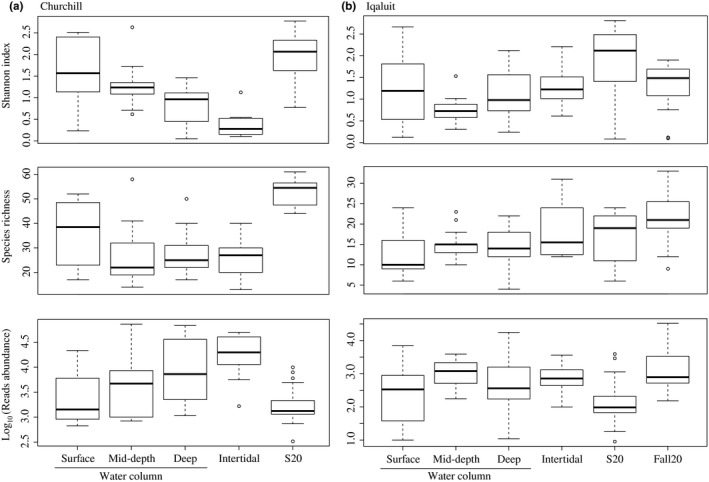
Boxplots comparing Shannon indices, species richness, and read abundances detected using eDNA metabarcoding for each sampling location (i.e., water column (surface, mid‐depth and deep), tide pools and S20 and Fall20) in Churchill and Iqaluit. The lines inside the boxes represents the median values, the top and bottom of the boxes represent the 75% and 25% quartiles and outliers are shown using empty circles (i.e., any data beyond 1.5*IQR)

The Shannon diversity index was significantly greater in the water column than tide pools in Churchill (ANOVA: *p *=* *0.002), but there was no significant difference in Iqaluit (*p *=* *0.2; Figure 5). In Churchill, despite a significantly greater number of reads in tide pools than the water column (averaging 23,276 and 11,623 reads in tide pools and water column samples, respectively; *p *=* *0.06), there was no significant difference in species richness between water column and tide pool samples (averaging 25.40 and 30.27 species in tide pools and water column samples, respectively; *p *=* *0.42; Figure 5). In contrast, in Iqaluit, despite the similar number of reads in the tide pool and water column samples (averaging 1,061 and 1,716 reads in tide pools and water column samples, respectively; *p *=* *0.50), species richness was significantly greater in tide pools than in the water column (averaging 18.33 and 13.92 species in tide pool and water column samples, respectively; *p *=* *0.02; Figure 5). In Iqaluit, the tide pools had estimated Sørenson similarity indices of 0.65, 0.64, 0.62 with the surface, mid‐depth and deep water, respectively, whereas in Churchill, the tide pools had slightly higher estimates of 0.67, 0.84, and 0.68 for the surface, mid‐depth and deep water, respectively.

**Figure 6:**
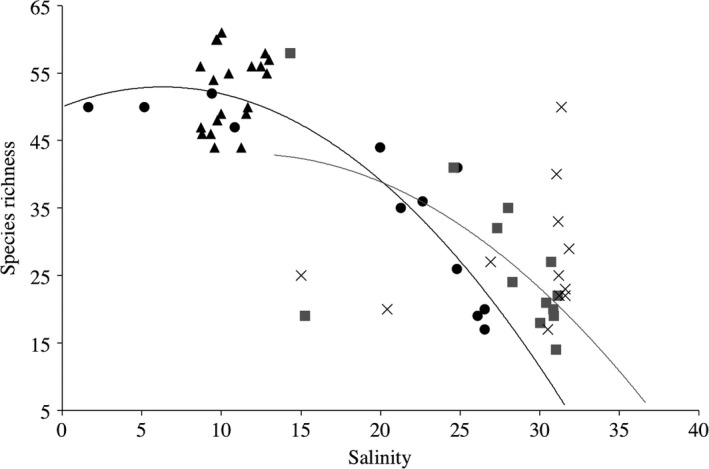
Relationship between the species richness detected using eDNA metabarcoding and the salinity of the water collected for the surface layer (*R* ^2^ = 0.85, black; circles: sampling water column and triangles: S20) and mid‐depth samples (*R* ^2^ = 0.44, gray squares) and deep water (gray cross)

The community structure differed significantly among the water depths, but the proportion of explained variance was greater for Churchill than Iqaluit (Figure 3, Churchill: *R* ^2^ = 0.13, *p *<* *0.001; Iqaluit: *R* ^2^ = 0.08, *p *=* *0.04), The Crustacean *Balanus balanus* dominated the deep water of both ports (cumulative contributions for Churchill = 80.0% mid‐depth vs. deep water and 67.1% surface vs. deep water; Iqaluit = 62.3% mid‐depth vs. deep water and 65.5% surface vs. deep water) and *Nemertea* was found only in mid‐depth in Iqaluit (Figure 5). In Iqaluit, the Shannon index, species richness and number of reads did not differ significantly among the depth layers (ANOVA shannon: *p *=* *0.1; species richness: *p *=* *0.3; reads abundance: *p *=* *0.1). In contrast, in Churchill, the Shannon index differed significantly among the depth layers (*p *≤* *0.001). Higher species richness was found at the surface (*p *=* *0.02), which generally corresponded to where there are more freshwater inputs from the Churchill River (Figure 6). Species detected only at the surface included 52.4% and 19.0% freshwater and brackish species, respectively. The mid‐depth similarity index was the highest among all water depth comparisons (Sørensen and Jaccard nonparametric estimates: 1.0 for Iqaluit and 0.92 for Churchill), but not significantly so relative to the Iqaluit surface‐deep and the Churchill intertidal‐mid, surface‐mid, and surface‐deep comparisons.

### 3.3 Seasonal variation

The community structure varied significantly between the summer and fall sampling (Figure 3, PERMANOVA; *R* ^2^ = 0.30, *p *<* *0.001); Arthropods dominated the summer samples, whereas Annelids dominated in fall (Figure 4) with a total of 54.1% shared species. Species richness was greater under ice cover than in summer (richness: *t *=* *2.3, *p *=* *0.02; Shannon index: *t* = −2.6, *p *=* *0.01), averaging 21 and 17 species in fall and summer samples, respectively (Figure 5).

## 4 DISCUSSION

Improved biodiversity monitoring programs are crucial for maintaining the integrity of coastal marine ecosystems. Evaluating the potential of eDNA to identify Arctic species and understanding the dynamics of eDNA distribution in coastal environments are both timely and important goals for improving biodiversity monitoring. Here, we present evidence that eDNA may be used to assess Arctic biodiversity and show that, despite the cold and well‐mixed environment, standardized eDNA approaches to biodiversity monitoring will need to consider local spatio‐temporal variation.

### 4.1 Taxonomic assignment challenges

The high congruence between historical Arctic data and eDNA samples (74.0%) supports the efficacy of aquatic eDNA metabarcoding for evaluating Arctic coastal biodiversity at the species level. The species detected with eDNA that were not previously known from the Canadian Arctic (42 species in Churchill and 11 species in Iqaluit) may be new species records, unexpected NIS or Arctic species that are not yet represented in the sequence reference databases that instead matched a closely related non‐Arctic species sequence. About 3,894–4,674 (4,284 ± 390) macro‐ and megabenthic species are estimated to inhabit the Arctic shelf regions (Piepenburg et al., 2011). However, Goldsmit et al. (2014) showed that approximately 15% of the taxa identified in Arctic ports were considered new records within the regions surveyed and approximately 8% within the more extensive adjacent surrounding regions. Piepenburg et al. (2011) suggested that further traditional sampling in the coastal Arctic would increase the number of Mollusca, Arthropoda and Echinodermata species by 26–52%, indicating that between about a fifth and a third of the expected Mollusca‐Arthropoda‐Echinodermata species pool is still unknown. Given these estimated biases in the historical data, it is therefore not surprising that the congruence between species detected by metabarcoding and historical data is not 100%.

A major shortcoming of metabarcoding is the incomplete state of reference sequence databases. Despite considerable barcoding efforts, reference sequences are still very limited for coastal benthic species, especially for remote regions such as the Arctic. Results showed that ~50% of known Arctic species are actually present in sequence databases and that a similar proportion of the eDNA sequences were assigned to species, indicating that reference database omissions are limiting eDNA metabarcoding surveys at this time and that COI sequencing efforts can rapidly improve Arctic biomonitoring. As shown by the low proportion of OTUs identified at the species level, Porifera and Rotifera were less likely to be detected than other groups such as Annelida (Figure 2). The use of eDNA metabarcoding may thus become a powerful approach to guide reference database improvement (e.g., 97% Rotifera OTUs were not identified at the species level). Moreover, groups such as Bryozoans, Nemerteans and Rotifera are currently not included in the historical Arctic Canada species records that we compiled, but they are important to coastal ecosystems and could be good indicators of biodiversity shifts caused by ice cover changes. The eDNA metabarcoding method might thus be a good practical approach to evaluate the community changes of such species groups, even when poorly identified at the species level. The better our knowledge of local species richness, potential invaders, and their corresponding genetic information, the more accurate our eDNA biodiversity monitoring methods will become. However, even when not assigned to species, the eDNA sequences detected here provide a sequence reference baseline that can be used to evaluate future species loss, new invasions, or other changes in community structure.

Once a taxon has been firmly identified by taxonomic experts and its barcode sequence has been deposited in GenBank or BOLD, eDNA might eventually reduce the need for large teams of expert taxonomists to carry out routine biodiversity monitoring. Yet, the routine application of metabarcoding for Arctic monitoring requires overcoming various limitations. For example, here the eDNA metabarcoding identified *Acartia tonsa*, a potential invader that has been previously recorded in the ecoregions of ports connected to Churchill (Chan et al., 2012). However, the current available COI sequences for *Acartia tonsa* form several distinct clades, some of which cluster with *Acartia hudsonica*, raising the possibility that the eDNA sequences assigned to *A. tonsa* actually belong to the native *A. hudsonica*. Thus, taxonomic expertise remains crucial for reducing biases of species distributions related to increasing use of large‐scale eDNA metabarcoding.

Using two COI primer pairs, we increased the level of genetic polymorphism recorded at the species level, thereby increasing the resolution of the method for biodiversity monitoring (Deagle, Jarman, Coissac, Pompanon, & Taberlet, 2014). In addition to increasing the number of species detected, combining multiple primers may also reduce bias of eDNA dominance among species groups (e.g., dominance shift between Arthropoda and Annelida; Figure 2). Despite the fact that the amplification of COI is often desirable to differentiate species using DNA barcoding procedures (Che et al., 2012), the degree of universality for COI primers is relatively low and so combining multiple COI primer pairs as we did enabled monitoring a greater proportion of the diversity. Further studies are, however, needed to evaluate how the combination of the primer sets may depict local species diversity. On the other hand, targeting genes with lower taxonomic specificity (e.g., 18S) could improve the detection of biodiversity shifts at higher levels (e.g., phyla level; see Bik et al., 2012; Deagle et al., 2014; Elbrecht & Leese, 2015).

Characterization of biodiversity with metabarcoding is biased at the amplification step (see Deiner et al., 2017; Freeland, 2017; Kelly et al., 2017 and Pawluczyk et al., 2015). Evaluating the primer bias of eDNA metabarcoding among primer pairs is currently limited due to the unknown nature of eDNA and actual technology used to characterize eDNA. Our selected primer pairs were previously tested on 104 zooplankton species and validated on mock metazoan communities collected in Canadian ports by Zhang (2017). However, even these in situ mock communities are not representative of the complex mixture of eDNA in real biological samples, as they consisted of purified DNA added in equimolar concentrations. Thus, future research evaluating the effects of primer bias is needed. Nevertheless, the results from our current comparisons show that there are important differences in eDNA community composition across space and time in samples collected using the same sampling and sequencing method. The large number of species detected in this study does allow for establishing a baseline for detecting species from their eDNA and measuring Arctic community structure changes. The current lack of knowledge on primer bias does limit comparisons of species lists and community structure between studies using different primer sets and genetic loci, however.

### 4.2 Spatio‐temporal eDNA variation

Our results clearly show that metazoan eDNA distribution in Arctic coastal environments has significant temporal and spatial variation. The transport of eDNA may be substantially higher compared to southern regions due to the limited degradation from cold water and the limited UV exposure during much of the year. Although eDNA is expected to be highly dispersed in cold environments, results here show clear horizontal and vertical eDNA heterogeneity in the Arctic. The observed heterogeneity of eDNA within and between samples suggests that, based on the summer and fall sample rarefaction curves, collecting at least 15 samples across as many sites as possible is optimal for comprehensive estimates of biodiversity variation (see Supporting Information Figure S1); an important metric for detecting effects of climate and shipping traffic change. A better understanding of the spatio‐temporal variation in eDNA due to local biotic and abiotic conditions will be important in standardizing comparisons of eDNA samples across spatial and temporal gradients in the Arctic marine environment.

Vertical eDNA distribution in the water column may vary as a function of the life cycle of species, transport and settling advection (Turner, Uy, & Everhart, 2015) and complex hydrodynamic processes. In addition to wave action on eDNA mixing (O’Donnell et al., 2017; Port et al., 2016), our data support the idea that in estuarine conditions, such as in Churchill, the freshwater flowing from the river over long distances may contribute to increasing the diversity in the surface water layer (e.g., Deiner & Altermatt, 2014; Jane et al., 2015). Community changes related to eDNA composition thus need to integrate information on temporal variation in river discharge. The variability in the eDNA capture zone should therefore combine complex interactions between community changes and hydrodynamic models.

The dominance of Mollusca reads in tide pools is consistent with the observed species composition in these habitats (e.g., Goldsmit, 2016). However, our results support the hypothesis that tides may modify differences in eDNA composition between the water column and tide pools. At the local scale, the eDNA distribution varied between habitats at both ports (i.e., water column and tide pools), but this pattern was more distinct in Churchill. The large tidal area in Iqaluit increases the water admixture between tide pools and the open ocean (11.72 m maximum tide in Iqaluit and 4.93 m in Churchill (Tide‐forecast 2017)), which may explain the relatively lower community differentiation between tide pool and water column sites in Iqaluit compared to Churchill.

Coastal biodiversity monitoring in the Arctic using traditional sampling approaches is generally limited to summer. In contrast to traditional surveys, the quality of eDNA surveys might actually improve under the ice cover due to the limited UV exposure and cold water temperature, hence promoting eDNA preservation and detection (Barnes et al., 2014). On the other hand, cold temperatures are expected to reduce the metabolism of species and associated eDNA release/detection (Lacoursière‐Roussel, Rosabal, et al., 2016). Here, eDNA metabarcoding of water collected under ice cover detected greater species richness than summer water collections. This is particularly relevant because the use of eDNA could expand the time window to survey coastal biodiversity in the Arctic. The observed species dominance changes between both seasons may also reflect life history (e.g., late Annelida reproduction; P. Archambault unpublished data). Here our survey is limited to two sampling periods, and thus further studies are needed to differentiate relative effects of species and eDNA ecologies between seasons (Hulbert, 1984).

### 4.3 Arctic conservation biology

As contributions of sequences from identified specimens increase to databases such as BOLD, so too will the ability to track biodiversity changes over time at the species level with powerful methods such as eDNA metabarcoding (Gibson et al., 2014; Ji, Ashton, & Pedley, 2013; Taylor & Harris, 2012). In the Arctic, the development of cost‐effective monitoring methods is essential for better protecting the integrity of important natural environments and endangered species and to ensure sustainable subsistence harvesting by aboriginal people, as well as recreational and commercial harvest by non‐Aboriginals. Applying eDNA metabarcoding to assess biodiversity in remote coastal regions offers several advantages toward increasing the speed and accuracy with which we can amass biodiversity data. As part of this research project, local community members and permanently stationed northern research staff were trained in eDNA sampling techniques with the goal of enabling a network of community‐based monitoring. In this context, we optimized eDNA strategies for remote regions. We first used a syringe method for filtering samples (Deiner & Altermatt, 2014), which allows for sampling at multiple sites simultaneously and limits cross‐contamination between samples as each sample can be processed with independent equipment. Moreover, the simplicity of this approach allows inexperienced collaborators to collect more eDNA samples per unit of time relative to standard practices of using an electric pump. Second, as storing and shipping frozen samples in remote regions is risky and often not possible, we used methods that allowed for DNA preservation at room temperature (Renshaw, Olds, Jerde, McVeigh, & Lodge, 2014). Lastly, the cost‐effective extraction method increases the ability to process large number of samples.

By overcoming methodological issues and improving knowledge about the ecology of eDNA in coastal area, this project creates the opportunity for future monitoring of metazoan coastal diversity in highly vulnerable ecosystems such as Arctic commercial ports. The combined benefits of being able to identify large numbers of species including local species and potential invaders, assess a large number of phyla, the local habitat variability and together with the effectiveness of the eDNA method under ice cover, are likely to make eDNA metabarcoding an efficient complementary approach to detect large‐scale Arctic coastal biodiversity changes. As the eDNA method progresses, the use of eDNA is likely to expand and become a catalyst for increased research on coastal biodiversity, ecosystem services, and sustainability, particularly in remote regions of the world such as the Canadian Arctic. However, spatio‐temporal dimensions need to be considered in standardizing and optimizing the assessment of marine biodiversity using eDNA.

## CONFLICT OF INTEREST

None declared.

## AUTHOR CONTRIBUTIONS

Anaïs Lacoursière‐Roussel is a conservation ecologist evaluating anthropogenic impacts on the dynamic of aquatic communities. All authors of this manuscript are interested developing and calibrating the eDNA method in the application of aquatic species distribution to improve the efficiency of conservation planning. ALR, LB, KH, PA, EG, and DL conceived the idea, ALR, LB, KH, KD, PA, and EN structured and edited the manuscript, KH and ALR developed the study design and participated in field collections. KH and PA are specialized in the Arctic coastal surveillance, CH, ALR, KD, and NL developed laboratory methodology and EN developed the analysis pipeline.

## DATA ACCESSIBILITY

Raw sequences reads were deposited in NCBI’s Sequence Read Archive (SRA, http://www.ncbi.nlm.nih.gov/sra) under Bioproject PRJNA388333.
