# Supplementary material for: eDNA metabarcoding as a new surveillance approach for coastal Arctic biodiversity
Source: Ecol Evol. 2018 Jul 13;8(16):7763–77. doi: 10.1002/ece3.4213 (PMC6144963; doi:10.1002/ece3.4213)
Supplement: Supplementary file 1 [file ECE3-8-7763-s001.docx]

**Supplementary files**

**eDNA metabarcoding as a new surveillance approach for coastal Arctic biodiversity**

Anaïs Lacoursière-Roussel^1^, Kimberly Howland^2^, Eric Normandeau^3^, Erin Grey^4^, Philippe Archambault^5^, Kristy Deiner^6^, David M. Lodge^7^, Cecilia Hernandez^3^, Noémie Leduc^3^ and Louis Bernatchez^3^

^1^ Fisheries and Oceans Canada, St. Andrews Biological Station (SABS), St. Andrews (New Brunswick), Canada, E5B 2L9

^2^ Fisheries and Oceans Canada, Central and Arctic Region, Freshwater Institute, Winnipeg (Manitoba), Canada, R3T 2N6,

^3^ Université Laval, Institut de Biologie Intégrative et des Systèmes (IBIS), Department of Biology, Québec (Québec), Canada, G1V 0A6

^4^ Governors State University, Division of Science, Mathematics and Technology, University Park (Illinois), USA, 60484

^5^  Université Laval, Québec-Océan, Department of Biology, Québec (Québec), Canada, G1V 0A6

^6^ Department of Evolutionary Biology and Environmental Studies, University of Zurich, 13 Winterthurerstr. 190, CH-8057 Zürich, Switzerland

^7^ Cornell University, Department of Ecology and Evolutionary Biology, Ithaca (New York), USA, 14853

**Appendix S1. eDNA extraction, amplification and sequencing**

To isolate and purify eDNA, 30 μl of Proteinase K (Qiagen) was added to the tubes containing the filter and the Longmire lysis buffer. Tubes were vortexed and incubated at 55**^◦^**C overnight. After incubation, the filter and lysis buffer mixture was centrifuged one minute at 13,000 RPM in a QIAshredder tube. 950 µl of the solution was then transferred to a new tube and 950 µl of the organic phase of phenol chloroform isoamyl alcohol (i.e. PCI, 25:24:1, Sigma P2069) was added. Tubes were hand shaken for five minutes and centrifuged for five minutes at 10,000 RPM. Supernatant was removed into a new tube and 950 µl of Chloroform-Isoamyl alcohol (CI, 24:1) was added to each tube. Tubes were then shaken for five minutes and centrifuged for five minutes at 10,000 RPM. 750 µl of the supernatant was transferred into a new tube and 750 µl of ice cold isopropanol and 375 µl of room temperature 5M NaCl were added to each tube and left overnight at -4^o^C. Tubes were centrifuged for 20 minutes at 13,000 RPM and isopropanol was then carefully poured off. 1,500 µl of cold Ethanol 70% was added and centrifuged for 20 minutes at 13,000 RPM. Ethanol was then carefully poured off and tubes were air dried with lid open in a laminar flow hood for 15 minutes. DNA was resuspended in 80 µl sterilized water (diH_2_0), placed in an incubator at 55°C for ten minutes and at 4°C overnight to resuspend the DNA. The extracted DNA was then frozen at -20 °C until amplification.

DNA amplifications were performed in a one-step dual-indexed PCR approach specifically designed for Illumina instruments by the “Plate-forme d’Analyses Génomiques” (IBIS, Université Laval). Two pairs of universal mitochondrial cytochrome c oxidase subunit I (COI) primers were used to amplify eDNA from as many metazoan taxa as possible: the forward mlCOIintF (Leray *et al.* 2013) and reverse jgHCO2198 (Geller et al. 2013) amplifying 313bp (hereafter called COI1) and the forward LCO1490 (Folmer *et al.* 1994) and reverse ill_C_R (Shokrella *et al.* 2015) amplifying 367bp (COI2). The primers were tailed on the 5’ end with part of the Illumina Nextera adaptors. The following adaptor sequence (regions that anneal to the flowcell and library specific barcodes) and oligonucleotide sequences were used for amplification: AATGATACGGCGACCACCGAGATCTACAC-[INDEX]-TCGTCGGCAGCGTCAGATGTGTATAAGAGACAG-[Forward primers] and reverse primers CAAGCAGAAGACGGCATACGAGAT-[INDEX]-GTCTCGTGGGCTCGGAGATGTGTATAAGAGACAG-[reverse primers]. Please note that primers used in this work contain Illumina specific sequences protected by intellectual property (Oligonucleotide sequences © 2007-2013 Illumina, Inc. All rights reserved. Derivative works created by Illumina customers are authorized for use with Illumina instruments and products only. All other uses are strictly prohibited).

Three PCR replicates were done for each sample and each primer set. The final reaction volume for each PCR replicate was 24 µL; including 12.5. µl Qiagen Multiplex Mastermix, 6.5 µl diH_2_0, 1 µl of each primer (10µM), and 3.0 µL of DNA. For all samples, the PCR mixture was denatured at 95°C for 15 min, followed by 35 cycles (94°C for 30 s, 54°C for 90 s (except for the COI2 primers, which was at 52°C for 90 s) and 72°C for 60s) and a final elongation at 72°C for 10 min. Products of the three aliquots were pooled for each sample. Because barcodes were different for each sample, a negative PCR control was done for each sample and primer set. All amplifications were visualized on a 1.5% agarose gel electrophoresis. If positive amplification of the PCR negative control was observed, amplification was redone with a newly diluted primer set. Because PCRs negative controls had the same barcode as the samples we could not pool them for sequencing. In contrast, field and extraction negative controls were treated exactly as regular samples and were independently indexed and pooled for sequencing. Pooled products were purified using Axygen PCR clean up kit following the manufacture’s recommended protocol. Libraries were quantified by AccuClear Ultra High Sensitivity dsDNA Quantitation Kit using the TECAN Spark 10M Reader for each sample and samples were pooled in equal molar concentrations to maximize equal sequence depth per sampling location (150 and 37 ng per site for COI1 and COI2 primer sets respectively in Churchill and 200 and 300 ng for COI1 and COI2 primer sets respectively in Iqaluit). When Quant-iT PicoGreen (Life Technologies) did not detect any DNA, 22.0 µL PCR mixtures were mixed nonetheless (see Table S3 for the concentration and volume for each sample separately).

Sequencing was carried out using an Illumina MiSeq (Illumina, San Diego, USA) at IBIS using a paired-end MiSeq Reagent Kit V3 (Illumina, San Diego, USA; sequence length = 300bp) and following the manufacturer’s instructions. For sequencing, the amplicon pool was diluted to 4 nM with molecular grade water, denatured and then sequenced at 10 pM following manufacturer’s instructions inclusive of spiking the samples with 15% of PhiX. Adaptor sequence and primer sequences were removed and raw sequencing reads de-multiplexed among samples using the MiSeq Control software v 2.3 into independent libraries. De-multiplexed reads were provided in gzip compressed Fastq format.

**Table S1.** *In vitro* validation of primers. Native species and potential invaders (low and high risk based on Chan et al. (2012), Chan et al. (2015) and Goldsmit (2016)) that primers were tested *in vitro*. ‘1’ depict a positive PCR amplification and ‘0’ that no amplification occurred.

| Species | Phylum | COI | |
| --- | --- | --- | --- |
|  |  | COI1 (mlCOIintF-jgHCO2198) | COI2( LCO149-illCR) |
| **Native** |  |  |  |
| *Gammarus oceanicus* | Arthropoda | 1 | 1 |
| *Astarte elliptica complexe* | Mollusca | 0 | 0 |
| *Musculus discors* | Mollusca | 0 | 0 |
| *Macoma calcarea* | Mollusca | 1 | 0 |
| *Hiatella arctica* | Mollusca | 0 | 1 |
| *Testudinalia testudinalis* | Mollusca | 1 | 0 |
| *Margarites groenlandicus* | Mollusca | 0 | 1 |
| *Margarites helicinus* | Mollusca | 1 | 0 |
| *Littorina saxatilis* | Mollusca | 1 | 1 |
| *Littorina obtusata* | Mollusca | 1 | 0 |
| *Macoma balthica* | Mollusca | 1 | 0 |
| *Mya truncata* | Mollusca | 1 | 0 |
|  |  |  |  |
| **Potential invader** |  |  |  |
| *Crassostrea gigas* | Mollusca | 0 | 0 |
| *Botryllus schlosseri* | Chordata (Tunicata) | 0 | 1 |
| *Ciona intestinalis* | Chordata (Tunicata) | 1 | 1 |
| *Styela clava* | Chordata (Tunicata) | 0 | 0 |
| *Jassa marmorata* | Arthropoda (amphipoda) | 1 | 1 |
| *Crepidula fornicata* | Mollusca | 1 | 0 |
|  |  |  |  |
| **Highly-potential invader** |  |  |  |
| *Caprella mutica* | Arthropoda (amphipoda) | 1 | 1 |
| *Littorina littorea* | Mollusca | 1 | 1 |
| *Botrylloides violaceus* | Tunicate | 0 | 1 |
| *Carcinus maenas* | Arthropoda (decapoda) | 1 | 0 |
| *Mya arenaria* | Mollusca | 1 | 0 |

**Appendix S2. References for native Arctic metazoans list include within the Fisheries and Oceans Canada Arctic Marine Invertebrate Database.**

Benthic Invertebrate References

1. Aitken AE, Gilbert R (1986) The biota of intertidal flats at Pangnirtung Fiord, Baffin Island, Northwest Territories. *Naturaliste can* **113**, 191–200.
2. Aitken AE, Risk MJ, Howard JD (1988) Animal-sediment relationships on a subarctic intertidal flat, Pangnirtung Fiord, Baffin Island, Canada. *Journal of Sedimentary Petrology* **58**, 969–978.
3. Atkinson EG, Wacasey JW (1989) Benthic invertebrates collected from Hudson Bay, Canada, 1953 to 1965. *Canadian Data Report of Fisheries and Aquatic Sciences* **744**, iv + 121 p.
4. Cusson M, Archambault P, Aitken A (2007) Biodiversity of benthic assemblages on the Arctic continental shelf: historical data from Canada. *Marine Ecology Progress Series* **331**, 291–304.
5. Ellis DV (1957) Marine infaunal benthos in Arctic North America. PhD thesis for the Faculty of graduate studies and research, Zoology, McGill University, Montreal, Canada.
6. Ellis DV (1960) Marine infaunal benthos in Arctic North America. *Arctic Institute of North America, Technical Paper N°5.*
7. Hopky GE, Lawrence MJ, McRae SM, Chiperzak DB (1994) List of Scientific names of Algae, Invertebrates and Vertebrates Captured under NOGAP Subprojects B.2.1 and B.2.3. 1984–1988. *Canadian Data Report of Fisheries and Aquatic Sciences* **924**, 76 pp.
8. ITIS (Integrated Taxonomic Taxonomic System) Available at http://www.itis.gov/.
9. Jirkov IA, Leontovich MK (2012) Biogeography of Polychaeta of the Aurasian North Polar Basin. *Invertebrate Zoology* **9**, 41–51.
10. Natural Museum. From P. Pocklington report.
11. North South Consultants (2006) Potential dispersal of aquatic invasive species into Hudson Bay from ballast water from ships travelling from ports in Europe and North America.
12. OBIS (Ocean Biogeographic Information system) Available at www.iobis.org.
13. Stewart PL, Pocklington P, Cunjak RA (1985) Distribution, Abundance and Diversity of Benthic Macroinvertebrates on the Canadian Continental Shelf and Slope of Southern Davis Strait and Ungava Bay. *Arctic* **36**, 281–291.
14. Thomson DH (1982) Marine benthos in the Eastern Canadian High Arctic: Multivariate analyses of standing crop and community structure. *Arctic* **35**, 61–74.
15. Thomson DH, Martin CM, Cross WE (1986) Identification and characterization of Arctic nearshore benthic habitats. *Canadian technical report of Fisheries and Aquatic Science N°1434.*
16. Samuelson GM (2001) Polychaetes as indicators of environmental disturbance on subarctic tidal flats, Iqaluit, Baffin Island, Nunavut Territory. *Marine Pollution Bulletin* **42**, 733–741.
17. SLB (Sea Life Base) Available at http://www.sealifebase.org/.
18. Stewart DB (2011) Species inhabiting the Tarium Niryutait Marine Protected Areas in the Canadian Beaufort Sea – Mackenzie Delta.
19. Wacasey JW, Atkinson EG, Glasspool L (1979) Zoobenthos data from upper Frobisher Bay, 1967–1973. *Canadian Data Report of Fisheries and Aquatic Sciences* **164**, 99.
20. Wacasey JW, Atkison EG, Glasspoole L, Bédard C (1980a) A marine biological study of Breevort Harbour and nearby waters of Eastern Baffin Island – Zoobenthos. *Canadian manuscript report of Fisheries and Aquatic Science N° 1557.*
21. Wacasey JW, Atkinson EG, Glasspoole L (1980b) Zoobenthos data from inshore stations of upper Frobisher Bay, 1969-1976. *Canadian Data Report of Fisheries and Aquatic Sciences* **205,** 42.
22. WoRMS (World Register of Marine Species) Available at www.marinespecies.org.

Zooplankton References

1. Andersen OGN (1981) Redescription of Marrus orthocanna (Kramp, 1942) (Cnidaria, Siphonophora). *Steenstrupia* **7**, 293–307.
2. Andronov VN, Kosobokova KN (2011) New species of small, bathypelagic calanoid copepods from the Arctic Ocean: Brodskius arcticus sp. nov. (Tharybidae) and three new species of Pertsovius gen. nov. (Discoidae). *Zootaxa* **2809**, 33–46.
3. Angel MV (1976) Bathyconchoecia arctica n. sp., a new species of ostracod (Halocyprididae, Myodocopidae) from the Arctic. *Crustaceana* **31**, 59–65.
4. ARCOD database. Available at http://www.arcodiv.org.
5. Audzijonyte A Väinölä R (2005) Diversity and distributions of circumpolar fresh- and brackish-water Mysis (Crustacea: Mysida): descriptions of M. relicta Lovén, 1862, M. salemaai n.sp., M. segerstralei n.sp. and M. diluviana n.sp., based on molecular and morphological characters. *Hydrobiologia* **544**, 88–141.
6. Auel H, Hagen W (2002) Mesozooplankton community structure, abundance and biomass in the central Arctic Ocean. *Marine Biology Berlin* **140**, 1013–1021.
7. Baker RF (1996) Abundance and species composition of zooplankton of the Nelson River estuary in relation to depth, tide, and location, July, 1995 - year 1. Unpublished report prepared by North/South Consultants Inc., Winnipeg, for Manitoba Hydro, Winnipeg, MB. viii + 65 p.
8. Bakker C (1980) On the distribution of ‘gonionemus vertens’ a. Agassiz (Hydrozoa, Limnomedusae), a new species in the eelgrass beds of Lake Grevelingen (S.W. Netherlands). *Aquatic Ecology* **14**, 186–195.
9. Bowman TE (1973) Pelagic amphipods of the genus Hyperia and closely related genera (Hyperiidea: Hyperiidae). *Smithsonian Contributions to Zoology* **136**, 1-76.
10. Bueckmann A (1969) Appendicularia. Fiches d'Identification du Zooplancton, 7: 1-9.
11. Cairns SD, Calder DR, Brinckmann-Voss A, Castro CB et al. (2002) Common and Scientific Names of Aquatic Invertebrates from the United States and Canada: Cnidaria and Ctenophora, Second Edition, 2002. American Fisheries Society Special Publication 28. xi + 115.
12. Choe N, Deibel D (2008) Temporal and vertical distributions of three appendicularian species (Tunicata) in Conception Bay, Newfoundland. J. *Plankton Research* **30**, 969-979.
13. Coad BW, Reist JD (2004) Annotated list of theArctic marine fishes of Canada. *Canadian Manuscript Report of Fisheries and Aquatic Sciences* **2674**, iv + 112p.
14. Coe WR (1956) Pelagic Nemertea: keys to Families and Genera. Fiches d'identification du zooplancton, 64. ICES: Copenhagen. 5 pp.
15. Cross W (1982) Under-ice biota at the Pond Inlet ice edge and adjacent fast ice areas during spring. *Arctic* **35**, 13–27.
16. Davis CC, Green JM (1974). Three monstilloids (Copepoda: Monstrilloida) from the Arctic. *Internationale Revue de gesamten Hydrobiologie und Hydrographie* **59**, 57–63.
17. Dunbar MJ (1942a) Marine macroplankton from the Canadian Eastern Arctic. I. Amphipoda and Schizopoda.—Canadian Journal of Research. Section D, *Zoological Sciences* **20**, 33–46.
18. Dunbar MJ (1942b) Marine macroplankton from the Canadian Eastern Arctic. II. Medusae, siphonophora, ctenophora, pteropoda, and chaetognatha. *Canadian Journal of Research* **20**, 71–77.
19. Dunbar MJ (1954) The amphipod Crustacea of Ungava Bay, Canadian eastern Arctic. *Journal of the Fisheries Research Board of Canada* **11**, 709–798.
20. Dunbar MJ (1963) Amphipoda. Sub-order Hyperiidea, Family Hyperiidae. *Fiches d'Identification du Zooplancton* **103**, 1–3.
21. Dunbar MJ (1964) Eupahusids and pelagic amphipods - Distribution in North Atlantic and Arctic waters. In: Serial atlas of the marine environment, Folio 6. American Geographical Society, New York, N.Y.
22. Evans MS, Grainger EH (1980) Zooplankton in a Canadian Arctic estuary, p. 199–210. In: V. S. Kennedy [Ed.]. Estuarine perspectives. Academic Press, New York, NY
23. Fenaux R, Bone Q, Deibel D (1998) Appendicularian distribution and zoogeography, p. 251-264. In q. Bone [ed.], The biology of pelagic tunicates. Oxford University Press.
24. Fishbase. Available at http://www.fishbase.org.
25. Forbes JR, Macdonald RW, Carmack EC, Iseki K, O'Brien MC (1992) Zooplankton retained by sequential sediment traps along the Beaufort Sea shelf break during winter. *Canadian Journal of Fisheries and Aquatic Science* **49**, 663–670.
26. Frost BW (1989) A taxonomy of the marine calanoid copepod genus Pseudocalanus. *Canadian Journal of Zoology* **67**, 525–551.
27. Gardner GA, Howell ET (1983) Zooplankton. Distribution across the shelf break of the southeast shoal of the Newfoundland Grand Banks in May 1981. *Canadian Manuscript Report of Fisheries and Aquatic Sciences* **1724**, iv + 61pp.
28. Gardiner K, Dick TA (2010) Arctic cephalopod distributions and their associated predators. *Polar Research* **29**, 209–227.
29. Grainger EH (1962) Zooplankton of Foxe Basin in the Canadian Arctic. *Journal of the Fisheries Research Board of Canada* **19**, 377–400.
30. Grainger EH (1965) Zooplankton from the Arctic Ocean and adjacent Canadian waters. *Journal of the Fisheries Research Board of Canada* **22**, 543–564.
31. Grainger EH (1975) Biological productivity of the Southern Beaufort Sea: the physical-chemical environment and the plankton. *Beaufort Sea Technical Report* **No. 12A**.
32. Grainger EH, Grohe C (1975) Zooplankton data from the Beaufort Sea, 1951 to 1975. *Canadian Fisheries Marine Service Technical Report* **591**, 1–54.
33. Harvey M, Starr M, Therriault JC, Saucier F, Gosselin M (2007) MERICA –Nord Program – monitoring and research in the Hudson Bay complex. Available at http://www.meds-sdmm.dfo-mpo.gc.ca/isdm-gdsi/azmp-pmza/docs/bulletin_5_07.pdf.
34. Hopcroft RR, Clarke C, Nelson RJ, Raskoff KA (2005) Zooplankton communities of the Arctic's Canada Basin: the contribution by smaller taxa. Polar Biology **28**, 198–206.
35. Hopky GE, Chiperzak DB, Lawrence MJ (1994a) NOGAP B2; Zooplankton, and larval and post larval fish data from Tuktoyaktuk Harbour, N.W.T., 1984 to 1987. *Canadian Data Report of Fisheries and Aquatic Sciences* **945**, v + 117 p.
36. Hopky GE, MJ Lawrence, Chiperzak DB (1994b) NOGAP B2; Zooplankton data from the Canadian Beaufort Sea shelf, 1987 and 1988. *Canadian Data Report of Fisheries and Aquatic Sciences* **912**, v + 219 p.
37. Hopky GE, Lawrence MJ, Chiperzak DB (1994c) NOGAP B2; Zooplankton data from the Canadian Beaufort Sea shelf, 1984 and 1985. *Canadian Data Report of Fisheries and Aquatic Sciences* **922**, iv + 164 p.
38. Hopky GE, Lawrence MJ, Chiperzak DB (1994d) NOGAP B2; Zooplankton data from the Canadian Beaufort Sea shelf, 1986. *Canadian Data Report of Fisheries and Aquatic Sciences* **923**, iv + 225 p.
39. Horner R, Murphy D (1985) Species composition and abundance of zooplankton in the nearshore Beaufort Sea in winter-spring. *Arctica* **38**, 201–209.
40. Jirkov IA (2001) [Polychaeta of the Arctic Ocean] Polikhety severnogo Ledovitogo Okeana. Moskva, Yanus-K, 1–632.
41. Kenchington ELR, Prena J, Gilkinson KD, Gordon DC Jr, MacIsaac K, Bourbonnais C, Schwinghamer PJ, Rowell TW, McKeown DL, Vass WP (2001) Effects of experimental otter trawling on the macrofauna of a sandy bottom ecosystem on the Grand Banks of Newfoundland. *Canadian Journal of Fisheries and Aquatic Sciences* **58**, 1043–1057.
42. Kosobokova KN, Hopcroft RR, Hirche H-J (2011) - Patterns of zooplankton diversity through the depths of the Arctic's central basins. *Marine Biodiversity* **41**, 29–50.
43. Kramp PL (1942) Siphonophora. The Godthaab Expedition 1928. *Meddeleser om Grønland* **80**, 3–24.
44. Kramp PL (1942) Medusae. The Godthaab Expedition 1928. *Meddeleser om Grønland* **81**, 1–168.
45. Maucheline J (1971) Euphausiacea adults. Fiches d'identification du zooplancton, 134. *ICES: Copenhagen*. 8 pp.
46. Levenstein RY (1981) [Some pecularities of the distribution of the family Polynoidae from the Canada basin of the Artic ocean]. Academy of Sciences of the USSR, Transactions of the P.P.Shirshov. *Institute of Oceanology* **115**, 26–36.
47. Markhaseva EL (2002) Phaennocalanus unispinosus (Copepoda, Calanoida, Phaennidae): new genus, and new species from the bathypelagial Arctic basin. *Sarsia* **87**, 312–318.
48. Mclaughlin *et al.* (2005) Common and scientific names of aquatic invertebrates from the United States and Canada: Crustaceans. American Fisheries Society, Special Publication 31, Bethesda, Maryland.
49. Mel'nikov IA, Kulikov AS (1983) The cryopelagic fauna of the central Arctic Basin [original in Russian]. Canadian Translation of Fisheries and Aquatric Sciences, 4910:1-92 (original publication 1980, Biol. Tsentral'nogo Arkticheskogo Basseina, p.97–111.
50. Muus BJ (1953a) Polychaeta, Families: Aphroditidae, Phyllodocidae and Alciopidae. Fiches d'identification du zooplancton, 52. *ICES: Copenhagen,* 6 pp.
51. Muus BJ (1953b) Polychaeta (contd.), Families: Tomopteridae and Typhloscolecidae. Fiches d'identification du zooplancton, 53. *ICES: Copenhagen*, 5 pp.
52. Ng ISY, Carr CM, Cottenie K (2009) Hierarchical zooplankton metacommunities: distinguishing between high and limiting dispersal mechanisms. *Hydrobiologia* **619**, 133–143.
53. North/South Consultants (2006) Potential dispersal of aquatic invasive species into Hudson Bay from ballast water from ships travelling from ports in Europe and North America. A report prepared for Fisheries and Ocean Canada File No. F2408-050083
54. Pepin P, Colbourne E, Maillet G (2011) Seasonal patterns in zooplankton community structure on the Newfoundland and Labrador Shelf. *Progress in Oceanography* **91**, 273–285.
55. Petryashov VV (2009) Order Mysidacea. In: Illustrated keys to the free-living invertebrates of Eurasian Arctic seas and adjacent deep waters. Vol. 1.: 65-83. Alaska Sea Grant College Program, University of Alaska Fairbanks.
56. Raskoff KA, Hopcroft RR, Kosobokova KN, Purcell JE, Youngbluth M (2010) Jellies under ice: ROV observations from the Arctic 2005 hidden ocean expedition. *Deep-Sea Research II*, 111-126.
57. Razouls C, de Bovée F, Kouwenberg J, Desreumaux N (2005-2015) - Diversity and Geographic Distribution of Marine Planktonic Copepods. Available at http://copepodes.obs-banyuls.fr/en.
58. Rogers GF (1981) Biological oceanography of Chesterfield Inlet, N.W.T., Summer 1978 – phytoplankton and zooplankton. Thesis, Univ. of Guelph, 185 pp.
59. Russell FS (1970) The Medusae of the British Isles. II. Pelagic Scyphozoa With a Supplement to the First Volume on Hydromedusae. Cambridge University Press, Cambridge. 284 pp.
60. Schuchert P (2001b) Survey of the family Corynidae (Cnidaria, Hydrozoa). *Revue Suisse de Zoologie* **108**, 739–878.
61. Schuchert P (2007) The European athecate hydroids and their medusae (Hydrozoa, Cnidaria): Filifera part 2. *Revue Suisse de Zoologie* **114**, 195–396.
62. Schuchert P (2010) The European athecate hydroids and their medusae (Hydrozoa, Cnidaria): Capitata Part 2. *Revue Suisse de Zoologie* **117**, 337–555.
63. Scott WB, Scott MG (1988) Atlantic Fishes of Canada. *Canadian Bulletin of Fisheries and Aquatic Sciences* **219**, 731p.
64. Shih CT (1977) A guide to the jellyfish of Canadian Atlantic waters.National Museum of Natural Scoences, Natural History Series, No.5, 90 pp.
65. Shih C-T, DR Laubitz (1978) Zooplankton distribution in the eastern Beaufort Sea and the Northwest Passage. *Astarte* **11**, 45–54.
66. Shih C-T, N Stallard (1982) Notes on two deep-water calanoids (*Aetideopsis rostrata* and *Neoscolecithrix farrani*) from Lancaster Sound. *Arctic* **35**, 56–60.
67. Shoemaker CR (1955) Amphipoda collected at the Arctic Laboratory, Office of Naval Research, Point Barrow, Alaska, by G. E. MacGinitie. *Smithsonian Miscellaneous Collections* **128**, 1–78.
68. Siferd TD, HE Welch, MA Bergmann, Curtis MF (1997) Seasonal distribution of sympagic amphipods near Chesterfield Inlet, N.W.T., Canada. *Polar Biology* **18**, 16–22.
69. Stephensen K (1933) The Godthaab Expedition 1928. Schizopoda. *Meddelelser om Grønland* **79**, 1–20.
70. Stübing D, Piepenburg D (1998) Occurrence of the benthic trachymedusa Ptychogastria polaris Allman, 1878 (Cnidaria: Hydrozoa) off Northeast Greenland and in the northern Barents Sea. *Polar Biology* **19**, 193–197.
71. Svavarsson J (1988) Bathyal and abyssal asollata (Crustacea, Isopoda) from the Norwegian, Greenland, and North Polar Seas. *Sarsia* **73**, 83–106.
72. Tattersall WM (1951) A review of the Mysidacea of the United States National Museum. *Bulletin of the United States National Museum* **201**, 1–292.
73. Tencati JR, Leung Y, Kobayashi H (1970) Taxonomic Guides to Arctic Zooplankton (I): Amphipods of the Central Arctic, Euphausiids of the Arctic Basin and Peripheral Seas. Technical rept. no. 2, 1959-1970, 61 pp.
74. Uchida T (1969) Medusae from the Arctic Ocean. *Publications of the Seto Marine Biological Laboratory* **17**, 285–287.
75. Walkusz W, Paulić JE, Kwaśniewski S, Williams WJ, Wong S, Papst MH (2010) Distribution, diversity and biomass of summer zooplankton from the coastal Canadian Beaufort Sea. *Polar Biology* **33**: 321–335.
76. Willey A (1920) Report on the marine Copepoda collected during the Canadian Arctic Expedition. Rep. *Canadian Arctic Expedition 1913-18* **7**, 1–46.
77. Willey A (1931) Biological and oceanographic conditions in Hudson Bay 4. Hudson Bay Copepod plankton. *Contributions Canadian Biology and Fisheries, N.S.* **6**, 483–493.
78. Woods SM, Smiley BD (1987) Arctic Data Compilation and Appraisal. Volume 9. Beaufort Sea: Biological Oceanography - Bacteria, Plankton, Epontic Community. Can. Data Rep. Hydrogr. *Ocean Science* **5**, 394 p.
79. WoRMS Editorial Board (2015). World Register of Marine Species. Accessed 2015-01-29. Available at http://www.marinespecies.org at VLIZ.
80. Zrum L (1999) Abundance and species composition of zooplankton in the Nelson River Estuary: baseline monitoring program 1998 – Year III. Unpublished report prepared by North/South Consultants Inc., Winnipeg, for Manitoba Hydro, Winnipeg, MB. viii + 71 p.
81. Zrum L (2000) Abundance and species composition of zooplankton in the Nelson River Estuary: baseline monitoring program 1999 – Year IV. Unpublished report prepared by North/South Consultants Inc., Winnipeg, for Manitoba Hydro, Winnipeg, MB. ix+ 74 p

**Appendix S3. Bioinformatic details**

Specific commands for preparation and analysis of the paired-end reads:

1. **Trim:** forward and reverse sense fastq files for each sample were trimmed using trimmomatic 0.30 with the following parameters: (TrimmomaticPE, -phred33, ILLUMINACLIP:"$ADAPTERFILE":3:30:6, LEADING:20, TRAILING:20, SLIDINGWINDOW:20:20, MINLEN:200 2).
2. **Merge**: cleaned reads were then merged using FLASH v1.2.11 with the following options: (-t 1 -z -O -m 30 -M 280). Only merged reads were used in the following analyses.
3. **Separate reads for each primer separately:** since merged sample files contained amplicons for four pairs of primers (two COI pairs and two 18s pairs), we split them each by amplicon using a Python script (split_amplicons_one_file.py). The script looks for degenerate primers at the beginning and end of each sequences and, in the case of positive identification for both primers, puts the sequence in the appropriate file.
4. **Chimeras**: regroup all the samples of one amplicon in a single file and use usearch with the -uchime_denovo command and the default parameters and the following output options: (-uchimeout, -chimeras, -uchimealns).

Specific commands to assign reads at the species level using the *Barque pipeline*, an open source and freely available metabarcoding analysis pipeline (www.github.com/enormandeau/barque):

1. **Sequences reference:** To maintain the phylum information, all animal BIN databases have been downloaded separately prior to formating. Separate files prior to formating have been pooled in the file names (eg: Chordata.fas.gz). Python script (format_bold_database.py) has been used to format each databases. Briefly, we retained only COI sequences that contained well formatted genus and species names. We also provided a file containing a list of unwanted species containing the names of all insect species. The formatted databases contain sequences whose names are in the phylum_Genus_species format. We then concatenated the formatted BOLD database into a single Fasta file and created a usearch compatible database usearch -makeudb_usearch command.
2. **Multiple hits: t**he presence of multiple hits has been assessed for some of the sequences with usearch using the usearch_local command with the following options: (-id 0.95, -maxaccepts 10, -maxrejects 50, -strand both -blast6out, -top_hits_only, -query_cov 0.5). We then ran the find_multiple_hits.sh script to parse the results and output a text file listing the cases of multiple hits and their frequency. The new unwanted species were added to the previous list of unwanted insect species, formatted and prepared the database again to remove them and used usearch with the usearch_local command with the following options to find the top hits only: (-id 0.9, -maxaccepts 6, -maxrejects 50, -strand both, -blast6out, -top_hit_only, -query_cov 0.5).
3. **Results** were summarized into phylum, genus and species count tables using the Python script 07_summarize_results.py with the following parameters: (min_similarity 97, min_length 300, min_coverage 1).

Specific commands to further explore missing species with the bold database using Operational taxonomic units (OTU) clustering:

1. **Read dereplication:** vsearch (--derep_fulllength --strand plus --sizeout --fasta_width 0)
2. **Operational taxonomic units (OTU) clustering:** OTUs were created using swarm 2.2.0 (-d 1, -f, -l,-w).
3. **Filtration:** OTUs represented by a single read (singletons) were excluded.
4. **Assignment:** the number of OTUs found with a ≤85% and ≤97% identity to a sequence in the BOLD database as well as the number of unique species retrived with at least 97% identity were found using vsearch (--usearch_global --qmask none --dbmask none --id 0.6 --blast6out --dbmatched --maxaccepts 20 --maxrejects 20 --maxhits 1 --query_cov 0.5 --fasta_width 0).
5. **Results** were summarized, for each phylum, site, and primer pair.

**Table S2.** Library preparation and bioinformatic pipeline details. Library details including the concentration (Conc., ng/µL) of the purified final PCR products measured by PicoGreen (ng/µL) and the final PCR volume mixed (µL) for each library (i.e. SRA accession number: SRX accession). Count of sequences for each data analysis step in the bioinfomatic pipeline (see Appendix S2) including the number of raw reads for the forward and reverse (i.e. count from the de-multiplexing), the reads remaining following trimming, merging (paired end runs), COI sequences within the expected amplicon length and without ambiguous nucleotides, without chimeras, and the sequences remaining with successful BLAST results (≥97% identity) and only for aquatic metazoan (removing insects, birds, algea and bacteria). Note that few non-aquatic species remained, but they were deleted before subsequent analyses. See the sampling site locations in Figure 1.

|  |  |  |  |  | Library | | | | |  | Bioinformatic pipeline | | | | | | | |
| --- | --- | --- | --- | --- | --- | --- | --- | --- | --- | --- | --- | --- | --- | --- | --- | --- | --- | --- |
| Port | Sample | SRA accession number | Sampling location | Sampling site | COI1 (mlCOIintF-jgHCO2198) | |  | COI2 (LCO1490-ill_C_R) | |  | Number of raw reads for the forward side | Number of raw reads for the reverse side | Trimmed reads forward | Trimmed reads Reverse | Number of raw reads following merging | COI Sequences remaining with expected length and without ambiguous nucleotides | Sequences remaining  without chimeras | Sequences with successful blast |
|  |  |  |  |  | Conc. | Vol. |  | Conc. | Vol. |  |  |  |  |  |  |  |  |  |
| Churchill | CH-11_S10 | SRR5658897 | surface | P2C | 77.71 | 1.93 |  | 5.86 | 11.05 |  | 304083 | 304083 | 275237 | 275237 | 274260 | 117984 | 117984 | 2045 |
| Churchill | CH-14_S34 | SRR5658898 | surface | P2B | 72.33 | 2.07 |  | 6.40 | 10.11 |  | 324054 | 324054 | 291625 | 291625 | 290668 | 94641 | 94641 | 22011 |
| Churchill | CH-17_S58 | SRR5658903 | surface | P8 | 75.07 | 2.00 |  | 13.93 | 4.65 |  | 281668 | 281668 | 253724 | 253724 | 252779 | 111175 | 111175 | 1589 |
| Churchill | CH-20_S11 | SRR5658894 | surface | P7 | 71.43 | 2.10 |  | 5.03 | 12.86 |  | 290629 | 290629 | 263324 | 263324 | 262464 | 112530 | 112530 | 1644 |
| Churchill | CH-21_S43 | SRR5658794 | surface | P9 | 72.15 | 2.08 |  | 14.29 | 4.53 |  | 255974 | 255974 | 232905 | 232905 | 232043 | 95948 | 95948 | 767 |
| Churchill | CH-24_S67 | SRR5658789 | surface | P4 | 79.40 | 1.89 |  | 35.33 | 1.83 |  | 286480 | 286480 | 258167 | 258167 | 257188 | 115048 | 115048 | 8099 |
| Churchill | CH-29_S44 | SRR5659067 | surface | P6 | 55.18 | 2.72 |  | 43.56 | 1.49 |  | 257483 | 257483 | 229346 | 229346 | 228586 | 85635 | 85635 | 1180 |
| Churchill | CH-4_S25 | SRR5659036 | surface | P3B | 75.22 | 3.20 |  | 22.31 | 2.90 |  | 280420 | 280420 | 255099 | 255099 | 253927 | 110392 | 110392 | 1985 |
| Churchill | CH-63_S63 | SRR5659127 | surface | P1B | 61.28 | 2.45 |  | 54.59 | 1.19 |  | 335859 | 335859 | 304633 | 304633 | 303687 | 128700 | 128700 | 1495 |
| Churchill | CH-64_S55 | SRR5659126 | surface | P1C | 56.73 | 2.64 |  | 77.63 | 0.83 |  | 369668 | 369668 | 336056 | 336056 | 335086 | 149025 | 149025 | 1327 |
| Churchill | CH-69_S15 | SRR5658835 | surface | P5 | 71.35 | 2.10 |  | 12.64 | 5.12 |  | 276458 | 276458 | 251307 | 251307 | 250380 | 112146 | 112146 | 5920 |
| Churchill | CH-7_S49 | SRR5659041 | surface | P10 | 76.39 | 1.96 |  | 1.31 | 20.00 |  | 269867 | 269867 | 242787 | 242787 | 241646 | 97353 | 97353 | 7076 |
| Churchill | CH-12_S18 | SRR5658896 | mid-depth | P2C | 51.13 | 2.93 |  | 1.10 | 20.00 |  | 241806 | 241806 | 215478 | 215478 | 213984 | 73443 | 73443 | 1223 |
| Churchill | CH-15_S42 | SRR5658901 | mid-depth | P2B | 62.30 | 2.41 |  | 0.17 | 20.00 |  | 206153 | 206153 | 181575 | 181575 | 180571 | 53026 | 53026 | 2710 |
| Churchill | CH-18_S66 | SRR5658902 | mid-depth | P8 | 91.63 | 1.64 |  | -0.07 | 20.00 |  | 245891 | 245891 | 217150 | 217150 | 215849 | 76825 | 76825 | 4570 |
| Churchill | CH-22_S51 | SRR5658787 | mid-depth | P9 | 80.27 | 1.87 |  | 52.70 | 1.23 |  | 263808 | 263808 | 223190 | 223190 | 222380 | 100793 | 100793 | 74820 |
| Churchill | CH-25_S4 | SRR5658790 | mid-depth | P4 | 71.61 | 2.09 |  | 29.24 | 2.21 |  | 307367 | 307367 | 275756 | 275756 | 274721 | 128933 | 128933 | 7622 |
| Churchill | CH-28_S36 | SRR5659068 | mid-depth | P6 | 64.61 | 2.32 |  | 8.39 | 7.72 |  | 285379 | 285379 | 255398 | 255398 | 253995 | 110049 | 110049 | 9399 |
| Churchill | CH-3_S17 | SRR5659037 | mid-depth | P3C | 90.97 | 1.65 |  | 5.49 | 11.79 |  | 247825 | 247825 | 222966 | 222966 | 221826 | 85507 | 85507 | 5216 |
| Churchill | CH-41_S27 | SRR5658792 | mid-depth | P7 | 76.40 | 1.96 |  | 6.89 | 9.40 |  | 238242 | 238242 | 202198 | 202198 | 200961 | 84956 | 84956 | 8909 |
| Churchill | CH-5_S33 | SRR5659043 | mid-depth | P3B | 76.61 | 2.85 |  | 32.56 | 1.99 |  | 255276 | 255276 | 230455 | 230455 | 229237 | 92707 | 92707 | 12974 |
| Churchill | CH-62_S71 | SRR5658737 | mid-depth | P1B | 59.22 | 2.53 |  | 29.13 | 2.22 |  | 302825 | 302825 | 275999 | 275999 | 275017 | 120872 | 120872 | 1275 |
| Churchill | CH-65_S47 | SRR5658843 | mid-depth | P1C | 68.73 | 2.18 |  | 14.28 | 4.53 |  | 257147 | 257147 | 231786 | 231786 | 230809 | 90495 | 90495 | 1197 |
| Churchill | CH-68_S23 | SRR5658838 | mid-depth | P5 | 88.68 | 1.69 |  | 3.31 | 19.57 |  | 281421 | 281421 | 255179 | 255179 | 253969 | 102410 | 102410 | 1020 |
| Churchill | CH-8_S57 | SRR5659040 | mid-depth | P10 | 96.13 | 1.56 |  | 25.99 | 2.49 |  | 255225 | 255225 | 222122 | 222122 | 221193 | 91489 | 91489 | 55880 |
| Churchill | CH-13_S26 | SRR5658899 | deep | P2C | 76.27 | 1.97 |  | 2.88 | 22.48 |  | 273525 | 273525 | 244825 | 244825 | 243692 | 90245 | 90245 | 11556 |
| Churchill | CH-19_S8 | SRR5658895 | deep | P1B | 51.94 | 2.89 |  | 4.61 | 14.05 |  | 280002 | 280002 | 250829 | 250829 | 249220 | 118388 | 118388 | 1436 |
| Churchill | CH-23_S59 | SRR5658788 | deep | P9 | 61.68 | 2.43 |  | 3.02 | 21.46 |  | 290468 | 290468 | 263435 | 263435 | 262154 | 128607 | 128607 | 31255 |
| Churchill | CH-26-1_S50 | SRR5658900 | deep | P2B | 85.64 | 1.75 |  | 6.02 | 10.76 |  | 354669 | 354669 | 316253 | 316253 | 314635 | 128376 | 128376 | 69823 |
| Churchill | CH-26-2_S20 | SRR5658786 | deep | P4 | 58.13 | 2.58 |  | 15.26 | 4.24 |  | 268010 | 268010 | 232266 | 232266 | 231238 | 96068 | 96068 | 43326 |
| Churchill | CH-27_S28 | SRR5659069 | deep | P6 | 49.33 | 3.04 |  | 4.27 | 15.15 |  | 322194 | 322194 | 290609 | 290609 | 288711 | 139401 | 139401 | 2283 |
| Churchill | CH-2_S9 | SRR5659038 | deep | P3C | 89.30 | 1.68 |  | 12.94 | 5.01 |  | 265413 | 265413 | 236444 | 236444 | 234432 | 91531 | 91531 | 6683 |
| Churchill | CH-42_S35 | SRR5658793 | deep | P7 | 42.50 | 3.53 |  | -0.81 | 20.00 |  | 207513 | 207513 | 185573 | 185573 | 184249 | 51514 | 51514 | 3871 |
| Churchill | CH-61_S3 | SRR5658738 | deep | P8 | 79.72 | 1.88 |  | 35.69 | 1.81 |  | 273227 | 273227 | 246720 | 246720 | 245912 | 99976 | 99976 | 2925 |
| Churchill | CH-66_S39 | SRR5658844 | deep | P1C | 67.71 | 2.22 |  | 4.88 | 13.26 |  | 260105 | 260105 | 234801 | 234801 | 233686 | 88421 | 88421 | 2442 |
| Churchill | CH-6_S41 | SRR5659042 | deep | P3B | 72.61 | 2.07 |  | 0.93 | 20.00 |  | 220835 | 220835 | 196403 | 196403 | 195110 | 64454 | 64454 | 9981 |
| Churchill | CH-9_S65 | SRR5659034 | deep | P10 | 83.48 | 1.80 |  | 38.65 | 1.68 |  | 265596 | 265596 | 235010 | 235010 | 233724 | 98079 | 98079 | 46257 |
| Churchill | CH-51_S72 | SRR5659010 | tide pool | P2A | 57.73 | 2.60 |  | 52.21 | 1.24 |  | 270325 | 270325 | 235101 | 235101 | 233809 | 106108 | 106108 | 43369 |
| Churchill | CH-52_S64 | SRR5659011 | tide pool | P2A | 34.36 | 4.37 |  | 18.94 | 3.42 |  | 254212 | 254212 | 222889 | 222889 | 221744 | 90799 | 90799 | 27349 |
| Churchill | CH-53_S56 | SRR5659125 | tide pool | P2A | 62.73 | 2.39 |  | 20.80 | 3.11 |  | 294228 | 294228 | 255763 | 255763 | 254218 | 114780 | 114780 | 43346 |
| Churchill | CH-54_S48 | SRR5659124 | tide pool | P2A | 54.74 | 2.74 |  | 31.23 | 2.07 |  | 251524 | 251524 | 218595 | 218595 | 217271 | 90518 | 90518 | 23285 |
| Churchill | CH-55_S40 | SRR5658742 | tide pool | P3A | 50.97 | 2.94 |  | 17.05 | 3.80 |  | 259916 | 259916 | 226694 | 226694 | 225371 | 93146 | 93146 | 16422 |
| Churchill | CH-56_S32 | SRR5658741 | tide pool | P3A | 24.36 | 6.16 |  | 24.89 | 2.60 |  | 242107 | 242107 | 211763 | 211763 | 210701 | 88403 | 88403 | 50841 |
| Churchill | CH-57_S24 | SRR5658740 | tide pool | P3A | 29.91 | 5.02 |  | 1.18 | 20.00 |  | 211122 | 211122 | 186085 | 186085 | 185138 | 65226 | 65226 | 11644 |
| Churchill | CH-58_S16 | SRR5658739 | tide pool | P3A | 35.89 | 4.18 |  | 5.71 | 11.35 |  | 280065 | 280065 | 248902 | 248902 | 248026 | 110727 | 110727 | 19047 |
| Churchill | CH-T76_S73 | SRR5659009 | tide pool | P1A | 22.92 | 6.54 |  | 9.06 | 7.15 |  | 254859 | 254859 | 223607 | 223607 | 222868 | 101021 | 101021 | 5880 |
| Churchill | CH-T80_S75 | SRR5659007 | tide pool | P1A | 41.72 | 3.60 |  | 9.48 | 6.83 |  | 270627 | 270627 | 246870 | 246870 | 246204 | 113428 | 113428 | 1828 |
| Churchill | CH-30_S62 | SRR5658842 | S20 |  | 60.53 | 2.48 |  | 37.25 | 1.74 |  | 316157 | 316157 | 281915 | 281915 | 280876 | 127631 | 127631 | 6690 |
| Churchill | CH-31_S52 | SRR5659066 | S20 |  | 60.89 | 2.46 |  | 66.09 | 0.98 |  | 289459 | 289459 | 256737 | 256737 | 255914 | 124154 | 124154 | 10954 |
| Churchill | CH-32_S60 | SRR5659073 | S20 |  | 64.34 | 2.33 |  | 30.07 | 2.15 |  | 294391 | 294391 | 264132 | 264132 | 263194 | 115087 | 115087 | 1877 |
| Churchill | CH-33_S68 | SRR5659072 | S20 |  | 59.15 | 2.54 |  | 23.39 | 2.77 |  | 329335 | 329335 | 295439 | 295439 | 294236 | 119763 | 119763 | 1712 |
| Churchill | CH-34_S5 | SRR5659071 | S20 |  | 36.60 | 4.10 |  | 35.43 | 1.83 |  | 266203 | 266203 | 239225 | 239225 | 238267 | 100788 | 100788 | 5486 |
| Churchill | CH-35_S13 | SRR5659070 | S20 |  | 61.93 | 2.42 |  | 18.87 | 3.43 |  | 287440 | 287440 | 257201 | 257201 | 256317 | 101609 | 101609 | 1032 |
| Churchill | CH-36_S29 | SRR5659074 | S20 |  | 60.51 | 2.48 |  | 47.79 | 1.35 |  | 163484 | 163484 | 118469 | 118469 | 117026 | 50396 | 50396 | 559 |
| Churchill | CH-37_S37 | SRR5658942 | S20 |  | 70.01 | 2.14 |  | 58.15 | 1.11 |  | 271497 | 271497 | 241571 | 241571 | 240711 | 106118 | 106118 | 1565 |
| Churchill | CH-38_S45 | SRR5658943 | S20 |  | 62.05 | 2.42 |  | 63.55 | 1.02 |  | 275186 | 275186 | 246189 | 246189 | 245304 | 94619 | 94619 | 1356 |
| Churchill | CH-39_S53 | SRR5658940 | S20 |  | 74.35 | 2.02 |  | 55.06 | 1.18 |  | 304096 | 304096 | 274851 | 274851 | 273916 | 124608 | 124608 | 1861 |
| Churchill | CH-40_S61 | SRR5658941 | S20 |  | 65.32 | 2.30 |  | 35.78 | 1.81 |  | 313520 | 313520 | 283189 | 283189 | 282254 | 125572 | 125572 | 2317 |
| Churchill | CH-43_S69 | SRR5658938 | S20 |  | 58.07 | 2.58 |  | 46.35 | 1.40 |  | 296365 | 296365 | 265609 | 265609 | 264643 | 113080 | 113080 | 8688 |
| Churchill | CH-44_S6 | SRR5658939 | S20 |  | 65.14 | 2.30 |  | 63.22 | 1.02 |  | 280096 | 280096 | 248653 | 248653 | 247714 | 101669 | 101669 | 1480 |
| Churchill | CH-45_S14 | SRR5658936 | S20 |  | 66.25 | 2.26 |  | 35.68 | 1.81 |  | 321896 | 321896 | 289431 | 289431 | 288381 | 118989 | 118989 | 1604 |
| Churchill | CH-46_S22 | SRR5658937 | S20 |  | 65.98 | 2.27 |  | 58.51 | 1.11 |  | 306955 | 306955 | 274497 | 274497 | 273454 | 114808 | 114808 | 2174 |
| Churchill | CH-47_S30 | SRR5658944 | S20 |  | 57.79 | 2.60 |  | 33.65 | 1.92 |  | 286115 | 286115 | 257932 | 257932 | 257042 | 101014 | 101014 | 1791 |
| Churchill | CH-48_S38 | SRR5658945 | S20 |  | 71.91 | 2.09 |  | 54.82 | 1.18 |  | 301879 | 301879 | 274344 | 274344 | 273472 | 115307 | 115307 | 1932 |
| Churchill | CH-49_S46 | SRR5658840 | S20 |  | 63.84 | 2.35 |  | 36.47 | 1.78 |  | 262865 | 262865 | 233175 | 233175 | 232329 | 83916 | 83916 | 3037 |
| Churchill | CH-50_S54 | SRR5658839 | S20 |  | 63.05 | 2.38 |  | 46.00 | 1.41 |  | 302460 | 302460 | 272847 | 272847 | 271879 | 119087 | 119087 | 1830 |
| Churchill | CH-70_S70 | SRR5658841 | S20 |  | 56.98 | 2.63 |  | 38.23 | 1.69 |  | 296604 | 296604 | 267705 | 267705 | 266781 | 115535 | 115535 | 1691 |
| Churchill | CH-T1_S2 | SRR5659033 | field control |  | -1.57 | 20.00 |  | -0.91 | 20.00 |  | 10778 | 10778 | 7319 | 7319 | 7253 | 319 | 319 | 68 |
| Churchill | CH-T2_S19 | SRR5658791 | field control |  | -1.82 | 20.00 |  | -1.85 | 20.00 |  | 16438 | 16438 | 13618 | 13618 | 13602 | 350 | 350 | 9 |
| Churchill | CH-T3_S12 | SRR5658785 | field control |  | -2.11 | 20.00 |  | -2.33 | 20.00 |  | 4982 | 4982 | 3086 | 3086 | 3069 | 189 | 189 | 8 |
| Churchill | CH-T4_S21 | SRR5659075 | field control |  | -1.98 | 20.00 |  | -1.90 | 20.00 |  | 6414 | 6414 | 4507 | 4507 | 4496 | 257 | 257 | 11 |
| Churchill | CH-T5_S7 | SRR5658836 | field control |  | -0.49 | 20.00 |  | -1.33 | 20.00 |  | 66076 | 66076 | 56691 | 56691 | 56539 | 5923 | 5923 | 163 |
| Churchill | CH-negA_S77 | SRR5658797 | extraction control |  | -1.56 | 20.00 |  | -1.45 | 20.00 |  | 3496 | 3496 | 1386 | 1386 | 1377 | 48 | 48 | 6 |
| Churchill | CH-negB_S78 | SRR5658798 | extraction control |  | -1.53 | 20.00 |  | -2.24 | 20.00 |  | 1030 | 1030 | 217 | 217 | 215 | 40 | 40 | 2 |
| Churchill | CH-negC_S79 | SRR5658803 | extraction control |  | -2.09 | 20.00 |  | -0.72 | 20.00 |  | 2499 | 2499 | 607 | 607 | 600 | 69 | 69 | 12 |
| Churchill | CH-negD_S80 | SRR5658804 | extraction control |  | -1.91 | 20.00 |  | -2.25 | 20.00 |  | 488 | 488 | 129 | 129 | 124 | 55 | 55 | 2 |
| Iqaluit | Iq-101_S67 | SRR5659005 | surface | P10 | 50.26 | 3.98 |  | 59.00 | 5.08 |  | 252040 | 252040 | 224417 | 224417 | 223884 | 83096 | 83096 | 1741 |
| Iqaluit | Iq-112_S60 | SRR5659110 | surface | P1B | 40.90 | 4.89 |  | 41.77 | 7.18 |  | 224744 | 224744 | 200101 | 200101 | 199649 | 78456 | 78456 | 85 |
| Iqaluit | Iq-109_S72 | SRR5659109 | surface | P1C | 46.85 | 4.27 |  | 43.37 | 6.92 |  | 157558 | 157558 | 140997 | 140997 | 140611 | 56204 | 56204 | 30 |
| Iqaluit | Iq-106_S55 | SRR5659102 | surface | P2B | 57.17 | 3.50 |  | 69.65 | 4.31 |  | 195743 | 195743 | 176091 | 176091 | 175676 | 78324 | 78324 | 928 |
| Iqaluit | Iq-103_S8 | SRR5659002 | surface | P2C | 45.36 | 4.41 |  | 52.21 | 5.75 |  | 160458 | 160458 | 143739 | 143739 | 143415 | 62816 | 62816 | 11 |
| Iqaluit | Iq-140_S64 | SRR5658784 | surface | P3B | 69.28 | 2.89 |  | 85.16 | 3.52 |  | 222426 | 222426 | 188990 | 188990 | 184864 | 70281 | 70281 | 7259 |
| Iqaluit | Iq-143_S45 | SRR5659025 | surface | P3C | 59.14 | 3.38 |  | 46.58 | 6.44 |  | 194700 | 194700 | 172040 | 172040 | 171469 | 60093 | 60093 | 421 |
| Iqaluit | Iq-122_S41 | SRR5659022 | surface | P4 | 65.41 | 3.06 |  | 54.09 | 5.55 |  | 222326 | 222326 | 197071 | 197071 | 196512 | 62165 | 62165 | 191 |
| Iqaluit | Iq-125_S23 | SRR5658988 | surface | P5 | 45.11 | 4.43 |  | 14.33 | 20.94 |  | 195855 | 195855 | 174917 | 174917 | 174299 | 47588 | 47588 | 341 |
| Iqaluit | Iq-119_S62 | SRR5658987 | surface | P6 | 47.01 | 4.25 |  | 51.16 | 5.86 |  | 201715 | 201715 | 180333 | 180333 | 179513 | 79041 | 79041 | 168 |
| Iqaluit | Iq-115_S59 | SRR5658982 | surface | P7 | 40.27 | 4.97 |  | 55.81 | 5.38 |  | 226991 | 226991 | 202491 | 202491 | 202145 | 79035 | 79035 | 687 |
| Iqaluit | Iq-128_S28 | SRR5659028 | surface | P8 | 56.00 | 3.57 |  | 57.72 | 5.20 |  | 173578 | 173578 | 156210 | 156210 | 155788 | 69512 | 69512 | 22 |
| Iqaluit | Iq-131_S53 | SRR5659031 | surface | P9 | 54.06 | 3.70 |  | 24.90 | 12.05 |  | 213189 | 213189 | 190672 | 190672 | 190181 | 69162 | 69162 | 2891 |
| Iqaluit | Iq-102_S35 | SRR5659004 | mid-depth | P10 | 31.32 | 6.39 |  | 23.10 | 12.99 |  | 184840 | 184840 | 160550 | 160550 | 160105 | 54854 | 54854 | 1639 |
| Iqaluit | Iq-113_S17 | SRR5659111 | mid-depth | P1B | 60.18 | 3.32 |  | 68.28 | 4.39 |  | 230499 | 230499 | 204857 | 204857 | 204355 | 70555 | 70555 | 249 |
| Iqaluit | Iq-110_S25 | SRR5659106 | mid-depth | P1C | 64.29 | 3.11 |  | 81.78 | 3.67 |  | 209699 | 209699 | 187067 | 187067 | 186687 | 76911 | 76911 | 4157 |
| Iqaluit | Iq-107_S26 | SRR5659103 | mid-depth | P2B | 21.91 | 9.13 |  | 52.49 | 5.72 |  | 251386 | 251386 | 228422 | 228422 | 228066 | 131165 | 131165 | 1854 |
| Iqaluit | Iq-104_S61 | SRR5659104 | mid-depth | P2C | 66.56 | 3.00 |  | 62.64 | 4.79 |  | 225147 | 225147 | 200353 | 200353 | 199858 | 95848 | 95848 | 2473 |
| Iqaluit | Iq-141_S57 | SRR5658783 | mid-depth | P3B | 41.96 | 4.77 |  | 29.58 | 10.14 |  | 172687 | 172687 | 155541 | 155541 | 155130 | 72004 | 72004 | 485 |
| Iqaluit | Iq-138_S24 | SRR5659026 | mid-depth | P3C | 44.15 | 4.53 |  | 41.19 | 7.28 |  | 159793 | 159793 | 140656 | 140656 | 140287 | 48020 | 48020 | 188 |
| Iqaluit | Iq-123_S54 | SRR5659023 | mid-depth | P4 | 57.68 | 3.47 |  | 50.34 | 5.96 |  | 230072 | 230072 | 206212 | 206212 | 205651 | 76817 | 76817 | 3236 |
| Iqaluit | Iq-126_S12 | SRR5658991 | mid-depth | P5 | 40.34 | 4.96 |  | 36.22 | 8.28 |  | 219181 | 219181 | 189646 | 189646 | 189243 | 60145 | 60145 | 1073 |
| Iqaluit | Iq-120_S3 | SRR5658986 | mid-depth | P6 | 52.54 | 3.81 |  | 60.45 | 4.96 |  | 238848 | 238848 | 212932 | 212932 | 212396 | 89410 | 89410 | 2745 |
| Iqaluit | Iq-116_S65 | SRR5658985 | mid-depth | P7 | 53.94 | 3.71 |  | 36.90 | 8.13 |  | 230554 | 230554 | 204818 | 204818 | 204268 | 73360 | 73360 | 535 |
| Iqaluit | Iq-129_S20 | SRR5659029 | mid-depth | P8 | 51.34 | 3.90 |  | 52.67 | 5.70 |  | 191758 | 191758 | 165394 | 165394 | 165010 | 52994 | 52994 | 681 |
| Iqaluit | Iq-132_S69 | SRR5658780 | mid-depth | P9 | 51.47 | 3.89 |  | 50.08 | 5.99 |  | 166006 | 166006 | 148888 | 148888 | 148580 | 64286 | 64286 | 1342 |
| Iqaluit | Iq-100_S85 | SRR5659003 | deep | P10 | 54.50 | 3.67 |  | 41.59 | 7.21 |  | 134167 | 134167 | 116274 | 116274 | 115883 | 51441 | 51441 | 3626 |
| Iqaluit | Iq-114_S22 | SRR5658983 | deep | P1B | 56.23 | 3.56 |  | 49.19 | 6.10 |  | 225276 | 225276 | 197713 | 197713 | 197166 | 66656 | 66656 | 422 |
| Iqaluit | Iq-111_S6 | SRR5659107 | deep | P1C | 47.92 | 4.17 |  | 32.15 | 9.33 |  | 214056 | 214056 | 187093 | 187093 | 186670 | 62668 | 62668 | 184 |
| Iqaluit | Iq-108_S2 | SRR5659108 | deep | P2B | 28.99 | 6.90 |  | 18.71 | 16.03 |  | 220824 | 220824 | 193034 | 193034 | 192532 | 58318 | 58318 | 1785 |
| Iqaluit | Iq-105_S70 | SRR5659105 | deep | P2C | 47.02 | 4.25 |  | 39.89 | 7.52 |  | 186276 | 186276 | 168545 | 168545 | 168088 | 76114 | 76114 | 290 |
| Iqaluit | Iq-142_S4 | SRR5658776 | deep | P3B | 41.15 | 4.86 |  | 67.36 | 4.45 |  | 243824 | 243824 | 213855 | 213855 | 213450 | 78897 | 78897 | 1671 |
| Iqaluit | Iq-139_S39 | SRR5659027 | deep | P3C | 47.13 | 4.24 |  | 55.28 | 5.43 |  | 144864 | 144864 | 131059 | 131059 | 130576 | 62942 | 62942 | 1033 |
| Iqaluit | Iq-124_S63 | SRR5659024 | deep | P4 | 57.32 | 3.49 |  | 38.20 | 7.85 |  | 184808 | 184808 | 162412 | 162412 | 161934 | 72117 | 72117 | 9922 |
| Iqaluit | Iq-127_S37 | SRR5658990 | deep | P5 | 68.97 | 2.90 |  | 32.59 | 9.21 |  | 148121 | 148121 | 130030 | 130030 | 129420 | 56361 | 56361 | 19 |
| Iqaluit | Iq-121_S44 | SRR5658989 | deep | P6 | 54.80 | 3.65 |  | 74.15 | 4.05 |  | 177794 | 177794 | 155212 | 155212 | 154613 | 55825 | 55825 | 18016 |
| Iqaluit | Iq-118_S11 | SRR5658984 | deep | P7 | 24.21 | 8.26 |  | 17.24 | 17.40 |  | 214428 | 214428 | 185291 | 185291 | 184614 | 50332 | 50332 | 27 |
| Iqaluit | Iq-130_S27 | SRR5659030 | deep | P8 | 52.45 | 3.81 |  | 60.79 | 4.94 |  | 176268 | 176268 | 147163 | 147163 | 146748 | 61223 | 61223 | 41 |
| Iqaluit | Iq-133_S32 | SRR5658779 | deep | P9 | 53.64 | 3.73 |  | 49.06 | 6.11 |  | 183341 | 183341 | 163821 | 163821 | 163478 | 60494 | 60494 | 329 |
| Iqaluit | Iq-134_S52 | SRR5658978 | tide pool | P2A1 | 43.43 | 4.61 |  | 81.80 | 3.67 |  | 250981 | 250981 | 215507 | 215507 | 214822 | 81188 | 81188 | 1502 |
| Iqaluit | Iq-135_S15 | SRR5658981 | tide pool | P2A2 | 51.55 | 3.88 |  | 59.89 | 5.01 |  | 235965 | 235965 | 207430 | 207430 | 206860 | 81372 | 81372 | 866 |
| Iqaluit | Iq-136_S9 | SRR5658980 | tide pool | P2A3 | 55.50 | 3.60 |  | 75.20 | 3.99 |  | 223808 | 223808 | 198735 | 198735 | 198146 | 80065 | 80065 | 3780 |
| Iqaluit | Iq-137_S19 | SRR5658973 | tide pool | P2A-4 | 46.44 | 4.31 |  | 50.39 | 5.95 |  | 207396 | 207396 | 182475 | 182475 | 182113 | 68567 | 68567 | 670 |
| Iqaluit | Iq-144_S18 | SRR5658972 | tide pool | P3A1 | 48.19 | 4.15 |  | 49.96 | 6.00 |  | 231667 | 231667 | 203373 | 203373 | 202899 | 77937 | 77937 | 2490 |
| Iqaluit | Iq-145_S13 | SRR5658968 | tide pool | P3A2 | 41.68 | 4.80 |  | 56.16 | 5.34 |  | 177493 | 177493 | 150996 | 150996 | 150538 | 60568 | 60568 | 323 |
| Iqaluit | Iq-146_S30 | SRR5658969 | tide pool | P3A3 | 53.34 | 3.75 |  | 36.70 | 8.17 |  | 162433 | 162433 | 139784 | 139784 | 139107 | 50944 | 50944 | 814 |
| Iqaluit | Iq-147_S50 | SRR5658970 | tide pool | P3A4 | 46.95 | 4.26 |  | 46.88 | 6.40 |  | 251498 | 251498 | 222378 | 222378 | 221654 | 79222 | 79222 | 1472 |
| Iqaluit | Iq-168_S92 | SRR5658971 | tide pool | P9A1 | 28.58 | 7.00 |  | 19.03 | 15.76 |  | 149788 | 149788 | 125786 | 125786 | 125401 | 29514 | 29514 | 418 |
| Iqaluit | Iq-169_S40 | SRR5658964 | tide pool | P9A2 | 44.86 | 4.46 |  | 54.77 | 5.48 |  | 170103 | 170103 | 148818 | 148818 | 148445 | 53777 | 53777 | 122 |
| Iqaluit | Iq-170_S14 | SRR5658965 | tide pool | P9A3 | 45.86 | 4.36 |  | 35.47 | 8.46 |  | 228590 | 228590 | 201881 | 201881 | 201320 | 74786 | 74786 | 898 |
| Iqaluit | Iq-171_S43 | SRR5658966 | tide pool | P9A4 | 46.46 | 4.30 |  | 46.73 | 6.42 |  | 139641 | 139641 | 125058 | 125058 | 124725 | 55035 | 55035 | 714 |
| Iqaluit | Iq-148_S33 | SRR5658775 | S20 |  | 25.77 | 7.76 |  | 20.10 | 14.93 |  | 150373 | 150373 | 134284 | 134284 | 134027 | 51972 | 51972 | 3029 |
| Iqaluit | Iq-149_S38 | SRR5658774 | S20 |  | 43.48 | 4.60 |  | 44.67 | 6.72 |  | 147882 | 147882 | 134450 | 134450 | 134021 | 53909 | 53909 | 100 |
| Iqaluit | Iq-150_S34 | SRR5658773 | S20 |  | 15.55 | 12.86 |  | 6.20 | 20.00 |  | 127989 | 127989 | 114601 | 114601 | 114237 | 29617 | 29617 | 12 |
| Iqaluit | Iq-151_S42 | SRR5658772 | S20 |  | 39.53 | 5.06 |  | 50.69 | 5.92 |  | 204940 | 204940 | 181078 | 181078 | 180719 | 64575 | 64575 | 507 |
| Iqaluit | Iq-152_S5 | SRR5658781 | S20 |  | 48.04 | 4.16 |  | 64.01 | 4.69 |  | 239878 | 239878 | 212161 | 212161 | 211726 | 82478 | 82478 | 178 |
| Iqaluit | Iq-153_S10 | SRR5658888 | S20 |  | 50.88 | 3.93 |  | 50.48 | 5.94 |  | 241089 | 241089 | 214193 | 214193 | 213694 | 77123 | 77123 | 105 |
| Iqaluit | Iq-154_S31 | SRR5658889 | S20 |  | 26.89 | 7.44 |  | 39.96 | 7.51 |  | 228105 | 228105 | 206273 | 206273 | 205677 | 74786 | 74786 | 80 |
| Iqaluit | Iq-155_S48 | SRR5658886 | S20 |  | 41.90 | 4.77 |  | 31.74 | 9.45 |  | 154754 | 154754 | 136649 | 136649 | 136315 | 46011 | 46011 | 155 |
| Iqaluit | Iq-156_S47 | SRR5658887 | S20 |  | 43.41 | 4.61 |  | 18.15 | 16.53 |  | 168259 | 168259 | 148600 | 148600 | 148203 | 43941 | 43941 | 76 |
| Iqaluit | Iq-157_S71 | SRR5658892 | S20 |  | 37.66 | 5.31 |  | 14.93 | 20.09 |  | 181335 | 181335 | 161850 | 161850 | 161447 | 51188 | 51188 | 50 |
| Iqaluit | Iq-158_S77 | SRR5658893 | S20 |  | 24.66 | 8.11 |  | 29.22 | 10.27 |  | 144767 | 144767 | 128158 | 128158 | 127738 | 43010 | 43010 | 85 |
| Iqaluit | Iq-159_S66 | SRR5658890 | S20 |  | 34.15 | 5.86 |  | 50.14 | 5.98 |  | 214319 | 214319 | 188353 | 188353 | 187764 | 76693 | 76693 | 1231 |
| Iqaluit | Iq-160_S21 | SRR5658891 | S20 |  | 51.68 | 3.87 |  | 47.66 | 6.29 |  | 218656 | 218656 | 194485 | 194485 | 193973 | 69922 | 69922 | 111 |
| Iqaluit | Iq-161_S68 | SRR5658880 | S20 |  | 50.42 | 3.97 |  | 54.81 | 5.47 |  | 228295 | 228295 | 203174 | 203174 | 202730 | 85438 | 85438 | 123 |
| Iqaluit | Iq-162_S29 | SRR5658881 | S20 |  | 43.96 | 4.55 |  | 52.68 | 5.69 |  | 102156 | 102156 | 67298 | 67298 | 66676 | 21649 | 21649 | 22 |
| Iqaluit | Iq-163_S36 | SRR5658975 | S20 |  | 44.17 | 4.53 |  | 61.31 | 4.89 |  | 221426 | 221426 | 197383 | 197383 | 196860 | 65322 | 65322 | 119 |
| Iqaluit | Iq-164_S58 | SRR5658974 | S20 |  | 41.21 | 4.85 |  | 50.23 | 5.97 |  | 258800 | 258800 | 230478 | 230478 | 229918 | 83002 | 83002 | 117 |
| Iqaluit | Iq-165_S49 | SRR5658977 | S20 |  | 46.58 | 4.29 |  | 46.51 | 6.45 |  | 217246 | 217246 | 194100 | 194100 | 193611 | 79025 | 79025 | 177 |
| Iqaluit | Iq-166_S81 | SRR5658976 | S20 |  | 51.11 | 3.91 |  | 39.71 | 7.55 |  | 192962 | 192962 | 170161 | 170161 | 169706 | 60544 | 60544 | 4040 |
| Iqaluit | Iq-167_S73 | SRR5658979 | S20 |  | 46.10 | 4.34 |  | 43.75 | 6.86 |  | 234646 | 234646 | 202562 | 202562 | 201889 | 71245 | 71245 | 375 |
| Iqaluit | Iq-T170_S7 | SRR5658816 | field control |  | 5.62 | 20.00 |  | 2.89 | 20.00 |  | 25958 | 25958 | 21074 | 21074 | 21026 | 7823 | 7823 | 31 |
| Iqaluit | Iq-T171_S46 | SRR5658823 | field control |  | 4.11 | 20.00 |  | 1.64 | 20.00 |  | 1888 | 1888 | 607 | 607 | 602 | 195 | 195 | 2 |
| Iqaluit | Iq-T172_S16 | SRR5658824 | field control |  | 4.55 | 20.00 |  | 6.48 | 20.00 |  | 10185 | 10185 | 7416 | 7416 | 7409 | 668 | 668 | 1 |
| Iqaluit | Iq-T173_S51 | SRR5658920 | field control |  | 4.27 | 20.00 |  | 2.12 | 20.00 |  | 4889 | 4889 | 3191 | 3191 | 3179 | 683 | 683 | 15 |
| Iqaluit | Iq-T174_S56 | SRR5658919 | field control |  | 5.04 | 20.00 |  | 7.13 | 20.00 |  | 42576 | 42576 | 33812 | 33812 | 33776 | 4543 | 4543 | 59 |
| Iqaluit | Iq-T175_S89 | SRR5658922 | field control |  | 1.43 | 20.00 |  | 6.13 | 20.00 |  | 49880 | 49880 | 41068 | 41068 | 40985 | 959 | 959 | 25 |
| Iqaluit | Iq-T176_S1 | SRR5658921 | field control |  | 4.58 | 20.00 |  | 1.93 | 20.00 |  | 8460 | 8460 | 6200 | 6200 | 6183 | 1849 | 1849 | 0 |
| Iqaluit | Iq-negA_S103 | SRR5658801 | extraction control |  | 5.82 | 20.00 |  | 5.67 | 20.00 |  | 2624 | 2624 | 608 | 608 | 604 | 36 | 36 | 1 |
| Iqaluit | Iq-negB_S76 | SRR5658802 | extraction control |  | 5.73 | 20.00 |  | 5.44 | 20.00 |  | 1870 | 1870 | 1312 | 1312 | 1312 | 187 | 187 | 4 |
| Iqaluit | Iq-negC_S80 | SRR5658795 | extraction control |  | 5.57 | 20.00 |  | 5.46 | 20.00 |  | 2066 | 2066 | 1215 | 1215 | 1215 | 82 | 82 | 3 |
| Iqaluit | Iq-negD_S84 | SRR5658796 | extraction control |  | 5.65 | 20.00 |  | 5.89 | 20.00 |  | 1184 | 1184 | 445 | 445 | 444 | 128 | 128 | 1 |
| Iqaluit | Iq-negE_S88 | SRR5658904 | extraction control |  | 5.69 | 20.00 |  | 5.68 | 20.00 |  | 2844 | 2844 | 2014 | 2014 | 2009 | 155 | 155 | 9 |
| Iqaluit | IqF-F1_S95 | SRR5658967 | F20 |  | 54.70 | 3.66 |  | 29.15 | 10.29 |  | 192490 | 192490 | 166238 | 166238 | 165347 | 59121 | 59121 | 2027 |
| Iqaluit | IqF-F10_S96 | SRR5659099 | F20 |  | 45.14 | 4.43 |  | 37.20 | 8.06 |  | 192231 | 192231 | 166480 | 166480 | 165880 | 57871 | 57871 | 29718 |
| Iqaluit | IqF-F11_S99 | SRR5659098 | F20 |  | 39.97 | 5.00 |  | 23.13 | 12.97 |  | 68009 | 68009 | 60093 | 60093 | 60055 | 53427 | 53427 | 5705 |
| Iqaluit | IqF-F12_S102 | SRR5659089 | F20 |  | 43.34 | 4.61 |  | 22.30 | 13.45 |  | 184325 | 184325 | 159916 | 159916 | 159188 | 45698 | 45698 | 844 |
| Iqaluit | IqF-F13_S75 | SRR5659088 | F20 |  | 47.98 | 4.17 |  | 29.07 | 10.32 |  | 208630 | 208630 | 180086 | 180086 | 179143 | 46320 | 46320 | 526 |
| Iqaluit | IqF-F14_S79 | SRR5658821 | F20 |  | 32.17 | 6.22 |  | 12.89 | 23.27 |  | 185048 | 185048 | 158156 | 158156 | 157446 | 30424 | 30424 | 1412 |
| Iqaluit | IqF-F15_S83 | SRR5658822 | F20 |  | 49.48 | 4.04 |  | 20.75 | 14.46 |  | 189226 | 189226 | 163335 | 163335 | 162620 | 47309 | 47309 | 218 |
| Iqaluit | IqF-F16_S87 | SRR5658819 | F20 |  | 30.33 | 6.59 |  | 13.86 | 21.65 |  | 161800 | 161800 | 139026 | 139026 | 138374 | 37423 | 37423 | 850 |
| Iqaluit | IqF-F17_S91 | SRR5658820 | F20 |  | 21.40 | 9.35 |  | 14.15 | 21.20 |  | 161755 | 161755 | 135282 | 135282 | 134647 | 27991 | 27991 | 362 |
| Iqaluit | IqF-F19_S94 | SRR5658818 | F20 |  | 37.97 | 5.27 |  | 23.29 | 12.88 |  | 157063 | 157063 | 133148 | 133148 | 132508 | 31824 | 31824 | 401 |
| Iqaluit | IqF-F2_S98 | SRR5658960 | F20 |  | 42.63 | 4.69 |  | 22.58 | 13.29 |  | 163230 | 163230 | 136359 | 136359 | 135831 | 40977 | 40977 | 4799 |
| Iqaluit | IqF-F20_S97 | SRR5658815 | F20 |  | 58.98 | 3.39 |  | 43.61 | 15.00 |  | 192105 | 192105 | 166470 | 166470 | 165759 | 56999 | 56999 | 33403 |
| Iqaluit | IqF-F28_S100 | SRR5658817 | F20 |  | 54.71 | 3.66 |  | 24.49 | 15.00 |  | 190777 | 190777 | 164079 | 164079 | 163524 | 60096 | 60096 | 1179 |
| Iqaluit | IqF-F3_S101 | SRR5658961 | F20 |  | 7.58 | 26.39 |  | 3.38 | 20.00 |  | 113748 | 113748 | 97960 | 97960 | 97396 | 17038 | 17038 | 172 |
| Iqaluit | IqF-F4_S74 | SRR5659097 | F20 |  | 33.04 | 6.05 |  | 25.64 | 11.70 |  | 180462 | 180462 | 156350 | 156350 | 155623 | 60473 | 60473 | 4862 |
| Iqaluit | IqF-F5_S78 | SRR5659096 | F20 |  | 51.04 | 3.92 |  | 21.83 | 13.74 |  | 181897 | 181897 | 156747 | 156747 | 156192 | 52576 | 52576 | 2939 |
| Iqaluit | IqF-F6_S82 | SRR5659095 | F20 |  | 34.39 | 5.82 |  | 13.17 | 22.78 |  | 169616 | 169616 | 145468 | 145468 | 144899 | 40058 | 40058 | 734 |
| Iqaluit | IqF-F7_S86 | SRR5659094 | F20 |  | 38.19 | 5.24 |  | 17.73 | 16.92 |  | 210834 | 210834 | 183220 | 183220 | 182386 | 58144 | 58144 | 781 |
| Iqaluit | IqF-F8_S90 | SRR5659101 | F20 |  | 57.70 | 3.47 |  | 41.18 | 7.29 |  | 227529 | 227529 | 198144 | 198144 | 197382 | 61072 | 61072 | 802 |
| Iqaluit | IqF-F9_S93 | SRR5659100 | F20 |  | 45.94 | 4.35 |  | 23.31 | 12.87 |  | 192197 | 192197 | 164600 | 164600 | 163831 | 40654 | 40654 | 681 |

**Table S3.** Reads with multiple species identified and actions taken in the pipeline based on the geographical species distributions.

| Reads | Location | Phylum | Species | Action |
| --- | --- | --- | --- | --- |
| 26 | Churchill | Mollusca | *Littorina saxatilis* | Keep |
|  |  |  | *Littorina compressa* | Delete |
|  |  |  | *Littorina arcana* | Delete |
|  |  |  |  |  |
| 19 | Churchill | Mollusca | *Littorina saxatilis* | Keep |
|  |  |  | *Littorina arcana* | Delete |
|  |  |  |  |  |
| 19 | Churchill | Echinodermata | *Strongylocentrotus pallidus* | Strongylocentrotus sp. |
| 22 | Iqaluit |  | *Strongylocentrotus droebachiensis* | Strongylocentrotus sp. |
|  |  |  |  |  |
| 14 | Churchill | Porifera | *Baikalospongia bacillifera* | Delete |
|  |  |  | *Swartschewskia papyracea* | Delete |
|  |  |  | *Lubomirskia baicalensis* | Delete |
|  |  |  | *Ephydatia muelleri* | Keep |
|  |  |  | *Baikalospongia recta* | Delete |
|  |  |  | *Baikalospongia intermedia* | Delete |
|  |  |  |  |  |
| 14 | Churchill | Chordata | *Coregonus nigripinnis* | Delete |
|  |  |  | *Coregonus artedi* | Keep |
|  |  |  | *Coregonus hoyi* | Delete |
|  |  |  | *Coregonus kiyi* | Delete |
|  |  |  | *Coregonus zenithicus* | Delete |
|  |  |  |  |  |
| 11 | Churchill | Mollusca | *Littorina saxatilis* | Keep |
|  |  |  | *Littorina compressa* | Delete |
|  |  |  |  |  |
| 4 | Churchill | Anthropoda | *Hypogastrura viatica* | Delete |
|  |  |  | *Hypogastrura purpurescens* | Delete |
|  |  |  |  |  |
| 3 | Churchill | Chordata | *Chen caerulescens* | Delete |
|  |  |  | *Chen rossii* | Delete |
|  |  |  |  |  |
| 1 | Churchill | Porifera | *Swartschewskia papyracea* | Delete |
|  |  |  | *porifera Spongilla lacustris* | Keep |
|  |  |  |  |  |
| 1 | Churchill | Porifera | *Ephydatia muelleri* | Keep |
|  |  |  | *Baikalospongia bacillifera* | Delete |
|  |  |  | *Swartschewskia papyracea* | Delete |
|  |  |  | *Lubomirskia baicalensis* | Delete |
|  |  |  | *Baikalospongia recta* | Delete |
|  |  |  | *Baikalospongia intermedia* | Delete |
|  |  |  |  |  |
| 1 | Churchill | Porifera | *Ephydatia fluviatilis* | Ephydatia sp. |
|  |  |  | *Ephydatia muelleri* | Ephydatia sp. |
|  |  |  | *Baikalospongia bacillifera* | Delete |
|  |  |  | *Swartschewskia papyracea* | Delete |
|  |  |  | *Lubomirskia baicalensis* | Delete |
|  |  |  | *Baikalospongia recta* | Delete |
|  |  |  | *Baikalospongia intermedia* | Delete |
|  |  |  |  |  |
| 1 | Churchill | Arthropoda | *Daphnia pulex* | Daphnia pulex |
|  |  |  | *Daphnia pulicaria* | Daphnia pulex |
|  |  |  |  |  |
| 7 | Iqaluit | Porifera | *Acanthorhabdus fragilis* | Porifera sp. |
|  |  |  | *Isodictya erinacea* | Porifera sp. |
|  |  |  | *Iophon unicorne* | Porifera sp. |
|  |  |  |  |  |
| 5 | Iqaluit | Chordata | *Lycodes seminudus* | Lycodes sp. |
|  |  |  | *Lycodes reticulatus* | Lycodes sp. |
|  |  |  | *Lycodes lavalaei* | Lycodes sp. |
|  |  |  |  |  |
| 4 | Iqaluit | Echinodermata | *Thyonidium drummondii* | Keep |
|  |  |  | *Thyone fusus* | Keep |
|  |  |  |  |  |
| 4 | Iqaluit | Arthropoda | *Gammarus oceanicus* | Gammarus sp. |
|  |  |  | *Gammarus setosus* | Gammarus sp. |

Figure S1. Rarefied species accumulation curves by read and sample numbers for each site for Churchill and Iqaluit. Read curves were plotted on the x-axis using the average number of reads per sample. Solid bold line denotes COI read rarefaction and light line denotes COI sample rarefaction. Errors bars represent 95% confidence intervals.

**Figure S2.** Species list and their known status within previous Canadian Arctic reported for each primers and port separately. The number of reads for each species detected from the eDNA (COI1 in blue and COI2 in red) collected in (A) Churchill and (B) Iqaluit in 2015. * indicate that this species was not previously detected in the Canadian Arctic, ** is a potential invader and ° only a single record of *Aurelia aurita*known from Canadian Arctic based on sequence data in BOLD from a partial specimen (from Churchill) that could not be morphologically identified to species level. Further research is needed to verify presence of this species in the Canadian Arctic.
